# Whole-Transcriptome Analysis of Three Human Cell Lines Stably Infected with Bovine Leukemia Virus (BLV) Reveals Novel Apoptosis-Associated Factors

**DOI:** 10.3390/v18090934

**Published:** 2026-08-27

**Authors:** Samy Metwally, Rania Hamada, Sonoko Watanuki, Ryosuke Matsuura, Yoko Aida

**Affiliations:** 1 Laboratory of Global Infectious Diseases Control Science, Graduate School of Agricultural and Life Sciences, The University of Tokyo, 1-1-1 Yayoi, Bunkyo-ku, Tokyo 113-8657, Japan; 2 Department of Infectious Diseases and Epidemics, Faculty of Veterinary Medicine, Damanhour University, Damanhour 22511, Egypt; 3 Department of Pathology and Clinical Pathology, Faculty of Veterinary Medicine, Damanhour University, Damanhour 22511, Egypt; 4 Laboratory of Global Animal Resource Science, Graduate School of Agricultural and Life Sciences, The University of Tokyo, 1-1-1 Yayoi, Bunkyo-ku, Tokyo 113-8657, Japan

**Keywords:** bovine leukemia virus (BLV), human cell lines, whole-transcriptome, RNA-seq, apoptosis pathways, *EFEMP1*, *LXN*

## Abstract

Bovine leukemia virus (BLV), a major cause of B-cell lymphoma in cattle worldwide, has been linked to human breast cancer. Here, we performed comparative whole-transcriptome analysis of single clones of human epithelial 293T, breast cancer MCF7, and cervical cancer HeLa cells, stably infected with BLV, versus uninfected controls using RNA-sequencing technology. Differential expression analysis revealed 2050, 1314, and 2772 differentially expressed genes (DEGs) between BLV-infected 293T, MCF7, and HeLa cells and controls, respectively. Most DEGs were upregulated in BLV-infected 293T (76.5%) and MCF7 (69.1%) cells but downregulated in HeLa cells (62.0%). Functional enrichment analyses revealed enrichment of “Gene expression” and “Membrane Trafficking” pathways across all cell lines, and that of “Apoptosis” and “Axon guidance” pathways uniquely in 293T and MCF7 cells. Twenty genes linked to apoptosis of 293T and MCF7 cells were identified, and expression of 11 of these was confirmed using quantitative real-time PCR. The expression of *EFEMP1* and *LXN*, which showed the greatest fold change, was validated: *EFEMP1* knockdown and *LXN* overexpression inhibited cell proliferation, induced apoptosis, and altered growth morphology of 293T and MCF7 cells. This is the first transcriptome profiling of human cells during BLV latency, which should help understand the behavior of BLV.

## 1. Introduction

Bovine leukemia virus (BLV), a member of the *Deltaretrovirus* genus, is the etiological agent of enzootic bovine leukosis, which is a chronic neoplastic disease marked by B-cell lymphoma in cattle [1,2]. BLV shares similarities with human T-cell leukemia virus type 1 (HTLV-1) in its genomic organization as well as gene expression and pathological strategies [2,3]. The virus is widely distributed worldwide, with 12 distinct genotypes circulating in cattle populations [4,5], and its global prevalence is increasing consistently [6]. Patterns of BLV spread differ substantially across regions and production systems [6]. Cattle with high proviral loads (HPVLs) are major sources of BLV spread [7,8]. BLV can be transmitted by blood-sucking insects, during iatrogenic practices that transfer infected blood between animals, and vertically from an infected dam to its fetus, intrapartum via contact with infected blood, or postpartum through infected milk [9]. BLV has also been detected in the milk, nasal secretion, and saliva samples of infected cows [10,11], beef cattle subjected to slaughter, and raw beef [12,13,14,15,16].

The natural BLV hosts include cattle, water buffaloes, and yaks [17,18]. Other animal species, such as sheep [19,20,21], goats [22], pigs [23], rabbits [24], and chickens [25], have been infected experimentally. The zoonotic potential of BLV has been reported in several studies [26], particularly following reports linking BLV infection to human breast cancer [27,28,29,30,31,32,33,34,35,36,37,38]. The presence of BLV has also been reported in human tissues and blood [39,40,41,42,43], and human cells are susceptible to BLV infection in vitro [21,30,44,45,46,47,48]. These studies have necessitated a molecular investigation for identifying the possible effects of BLV infection and persistence on the host transcriptomic architecture. Contrary to the above-mentioned reports on the presence of BLV in humans, BLV was believed to be nontransmissible to humans because several researchers from some BLV-endemic countries could not detect BLV in human samples [26,28,49].

In recent years, the transcriptomic response to BLV infection has been extensively studied in infected cattle [50,51,52,53,54,55], bovine mammary epithelial cell line (MAC-T) [56], and BLV-infected sheep [57] using high-throughput RNA sequencing (RNA-seq) analysis. These studies have provided an understanding of the responses that occur during different stages of BLV infection. Using RNA-seq, Nishimori et al. (2023) demonstrated upregulation of host genes during enzootic bovine leukosis progression, independent of the overexpression of viral transcriptional regulators [52]. Scull et al. (2024) analyzed the transcriptomes of peripheral blood mononuclear cells (PBMCs) of cows seropositive and seronegative for BLV antibodies and reported major differences in transcriptome profiles and suppression of immune responses caused by BLV infection [53]. González-Méndez et al. (2025) demonstrated that innate and cellular immune responses are more loose under conditions of severe BLV infection [54]. Similarly, a comparative RNA-seq analysis of cattle with contrasting proviral loads revealed downregulation of immune response genes in cows with HPVLs [55]. Based on RNA-seq analysis of blood samples from five calves experimentally infected with BLV, Bai et al. (2020) demonstrated that BLV infection affects host genes associated with DNA mismatch repair, offering new candidate markers for lymphoma diagnosis [51]. In a bovine cell culture model, BLV infection altered the expression of interferon I (IFN I) signal pathway and shifted the expression of proto-oncogenes and tumor suppressor genes in MAC-T cells [56]. Ashrafi et al. (2021) identified common genes between BLV and HTLV-1 using RNA-seq data generated from PBMCs of BLV-infected sheep [57]. In addition, microarray-based gene expression analysis showed that Tax protein, which contributes to the oncogenic potential of BLV, activates the expression of many cellular genes, encoding proteins such as activator protein 1 (AP-1) signaling pathway-related proteins (FBJ osteosarcoma oncogene (FOS), jun proto-oncogene (JUN), etc.) via interactions with other transcriptional pathways (G-proteins, GTP-binding proteins, etc.) and immune response-related proteins [50].

To date, the mechanisms underlying BLV interaction in human cells remain poorly understood, and no genome-wide transcriptomic profile is available in the literature. The lack of stable in vitro models for studying BLV–human cell interactions has hindered the understanding of the behavior of this virus in human cells. This gap in understanding is further complicated by new evidence suggesting that BLV engages an unknown mechanism in cancer patients different from that employed in infected cattle [58]. Additionally, Jaworski [58] reported the absence of BLV miRNA in human cancer small RNA-seq datasets, casting substantial doubt on the zoonotic potential of BLV. To address this gap, we previously established and characterized three human cell lines—human epithelial 293T, human cervical cancer HeLa, and human breast cancer MCF7—stably infected with BLV over an 18-month period [59]. In all these cell lines, BLV latency was associated with silencing, whereas the virus induced apoptosis and morphological changes in 293T and MCF7 cells [59]. In the present study, we performed a comparative whole-transcriptome RNA-seq analysis of the three human cell lines stably infected with BLV versus their uninfected controls. The study findings provide new insights into the potential for BLV latency to modulate the intracellular dynamics of gene expression profiles and biological pathways involved. Moreover, differential expression analysis enabled us to identify host genes related to apoptosis that are induced by BLV latency in human cells. Overall, our results provide an understanding of the differences between cells in which BLV induces morphological changes and apoptosis and those in which it does not induce such changes, offering clues for BLV pathogenesis in humans.

## 2. Materials and Methods

### 2.1. Cell Lines and Culture

Human embryonic kidney epithelial 293T cells (293T), human breast cancer MCF7 cells (MCF7) (ATCC HTB-22^TM^) (ATCC, Manassas, VA, USA), and human cervical cancer HeLa cells (HeLa) were used as negative uninfected parental controls. Single clones of previously established 293T (293T-BLV), MCF7 (MCF7-BLV), and HeLa (HeLa-BLV) cells stably infected with BLV as representative epithelial, breast cancer, and cervical cancer models [59] were used in this study. BLV existence and integrity in these clones were previously confirmed by BLV full-length PCR (≃8 kb), partial *env* gene nested PCR, and CoCoMo-qPCR targeting long terminal repeats (LTRs), with the BLV PVLs being 2730 copies per 150 ng of DNA for 293T-BLV, 3500 copies per 150 ng of DNA for MCF7-BLV, and 6180 copies per 150 ng of DNA for HeLa-BLV [59]. However, BLV silencing was observed, as evidenced by a lack of cellular expression of viral gp51 and p24 proteins, cell-to-cell infectivity, or detectable virion p24 and RNA throughout the study [59]. Some characteristics of these single clones are listed in Table 1. All cell lines were cultured in Dulbecco’s modified Eagle’s medium (DMEM) (Thermo Fisher Scientific, Waltham, MA, USA) supplemented with 10% heat-inactivated fetal bovine serum (Sigma-Aldrich, St. Louis, MO, USA), non-essential amino acids (Thermo Fisher Scientific), and penicillin–streptomycin–glutamine (Thermo Fisher Scientific) at 37 °C in a 5% CO_2_ atmosphere, and serially passaged upon approaching confluence for 18 months. For RNA-seq analysis, after the cell lines were passaged 100 times, three confluent 10 mL culture plates of each BLV-infected cell line or uninfected parental control cells (*n* = 6) were used for RNA extraction (*n* = 18). Independent culture replicates in each group were subjected to separate RNA extractions, yielding 18 RNA samples.

### 2.2. Total RNA Extraction and Quality Testing

Confluent cell cultures in 10 mL culture plates were washed twice with phosphate-buffered saline (PBS) and harvested with 300 µL of 0.25% trypsin-ethylenediaminetetraacetic acid (EDTA) (Thermo Fisher Scientific). Total RNA was extracted from 2 × 10^6^ cells using the NucleoSpin^®^ RNA isolation kit (MACHEREY-NAGEL GmbH & Co., Düren, Germany), according to the manufacturer’s instructions. RNA concentration and purity (Supplementary Table S1) were measured with a NanoDrop One spectrophotometer (Thermo Fisher Scientific) and Qubit^®^ 2.0 Fluorometer (Life Technologies Co., Carlsbad, CA, USA) using a Qubit^TM^ RNA HS buffer (Thermo Fisher Scientific), according to the manufacturer’s instructions.

### 2.3. RNA Sequencing and RNA-Seq Data Analysis

RNA sequencing was outsourced to Novogene Corporation (NovogeneAIT Genomics Singapore Pte. Ltd. Helios, Singapore) (https://www.novogene.com/amea-en/, accessed on 10 July 2025). All RNA samples passed the quality control parameters of NovogeneAIT Genomics (NovogeneAIT Genomics Singapore Pte. Ltd.) and were sequenced using a directional mRNA poly-A enrichment library on an Illumina NovaSeq™ X Plus system (Illumina, San Diego, CA, USA) with a paired-end 150 bp sequencing. A minimum of 30 million reads per sample (approximately 9 G of raw data) was targeted.

RNA-seq data provided by Novogene Corporation were analyzed using the RaNA-seq web platform (https://ranaseq.eu). A full RNA-seq analysis was performed using the bioinformatics and statistical tools integrated in this platform, as described previously [60]. Data from each BLV-infected cell line were analyzed against its corresponding uninfected, parental control cell line (i.e., 293T-BLV vs. 293T uninfected, MCF7-BLV vs. MCF7 uninfected, and HeLa-BLV vs. HeLa uninfected). Briefly, FASTQ RNA-seq data files were subjected to quality assessment and adaptor trimming using the Fastp tool [61], followed by mapping to the Genome Reference Consortium Human Build 38 (GRCh38) using Salmon [62]. The aligned transcripts were quantified and quality control reports were generated. Counts were normalized as transcripts per million, and principal component analysis (PCA) plots and heatmaps were generated using the RJSplot package [63]. Distances between each pair of samples were calculated with SERE [64]. Differential expression analysis was performed with the DESeq2 tool in R (version 3.5.2) [65], followed by multiple comparison correction of the *p*-values using the Benjamini–Hochberg false discovery rate method [66]. The differentially expressed genes (DEGs) were identified based on the following criteria: adjusted *p*-value < 0.05 and a log_2_ fold-change of at least 1 or −1 [54]. Finally, the functional enrichment of DEGs was performed using the annotation database generated in the RaNA-seq platform, which integrates gene ontology (GO) analysis and pathway annotations from the National Center for Biotechnology Information (NCBI) BioSystems. REACTOME and Kyoto Encyclopedia of Genes and Genomes (KEGG) databases were used for pathway analysis. The volcano plots and networks were generated with various packages integrated in the RaNA-seq platform [60]. Subsequently, the total identified DEGs were filtered strictly based on the expression criteria (|log_2_FC| ≥ 1.5 and *p*-adjust < 0.01). Then, we selected genes that showed consistent directional expression changes in both 293T-BLV and MCF7-BLV cells, while remaining unchanged or exhibiting a different expression pattern in HeLa-BLV cells.

### 2.4. Reverse Transcription–Quantitative PCR (RT-qPCR)

For validation of RNA-seq analysis, mRNA levels of 15 selected genes and the housekeeping gene, glyceraldehyde 3-phosphate dehydrogenase (*GAPDH*), were quantified via RT-qPCR. Briefly, 2 μg of RNA from each sample was reverse transcribed into cDNA using the High-Capacity RNA-to-cDNA Kit (Applied Biosystems, Foster City, CA, USA) according to the manufacturer’s instructions. The primer sequences (Supplementary Table S2) were either derived from previous studies [67,68,69,70,71,72,73,74,75] or designed using the NCBI primer-designing tool. RT-qPCR was conducted using KAPA SYBR^®^ FAST qPCR Kit (KAPA BIOSYSTEMS, Wilmington, MA, USA) on an Applied Biosystems 7500 Fast Real-Time PCR system (Applied Biosystems). The thermocycling conditions were as follows: 95 °C for 3 min, followed by 40 cycles of 95 °C for 10 s and 60 °C for 30 s. Prior to RT-qPCR data analyses, *GAPDH* expression stability was verified across all uninfected cell lines and BLV-infected cells by comparing the raw CT values across independent replicates, showing no statistically significant differences (*p* > 0.05). Each sample was tested in duplicate, and the data were analyzed using the comparative CT method (∆∆CT) with normalization against *GAPDH* mRNA expression [76].

### 2.5. Western Blot Analysis

Cells were washed twice with PBS and harvested with 0.25% trypsin-EDTA (Thermo Fisher Scientific). The harvested cells were lysed for 30 min on ice in 20 mM Tris-HCl (pH 7.4), 300 mM NaCl, 2 mM EDTA, and 2% NP40 supplemented with a protease inhibitor cocktail (Roche Diagnostics, Mannheim, Germany). The cell lysates (30 µg) were subjected to Western blotting using rabbit polyclonal EFEMP1/Fibulin-3 antibody (Abcam, Cambridge, UK), rabbit polyclonal LXN/TCI antibody (Abcam), or anti-β-actin MAb (Sigma-Aldrich), as described previously [59,77].

### 2.6. EFEMP1 siRNA Knockdown

A predesigned siRNA for targeting human EFEMP1 (EFEMP1 siRNA) was constructed by Silencer Select siRNAs (Ambion, Austin, TX, USA). EFEMP1 was knocked down in 293T and MCF7 cells using Silencer^TM^ Select Product # s5050. Scramble siRNA negative control (vector siRNA) was obtained from Ambion (catalog# 4390843). A day before transfection, 293T (5 × 10^5^) and MCF7 (1 × 10^6^) cells were seeded in 6-well plates and incubated at 37 °C in a 5% CO_2_ atmosphere. The next day, the cells were transfected with vector or EFEMP1 siRNA using the Lipofectamine RNAiMAX reagent (Thermo Fisher Scientific) according to the manufacturer’s instructions. To evaluate the knockdown efficiency, cell lysates were prepared and subjected to RT-qPCR or Western blot analysis at 48 or 72 h post-transfection, respectively [76].

### 2.7. Construction of LXN Overexpression Plasmid and Transfection

A previously constructed [78] pME18neo-Flag-IRES-ZsGreen1 control vector (NC Vector), containing IRES and ZsGreen1 coding sequences, was used as a template for constructing pME18neo-LXN-IRIS-ZsGreen1 (LXN-plasmid), which was used to overexpress human LXN. Briefly, the *LXN* open reading frame (669 bp fragment) was amplified from 293T and MCF7 cDNAs using the primers 5′-ATGGAAATCCCGCCGACCAAC-3′ and 5′-TTATTCCAGTTGTACTTCCTTTGG-3′ with Prime STAR GXL DNA Polymerase (Takara Bio Inc., Kusatsu, Japan) according to the manufacturer’s instructions. The PCR fragment was further amplified with 15 bp extension primers homologous to vector ends (5′-GCGGAATTCCTCGAGATGGAAATCCCGCCGACCAA-3′ and 5′-TTCCAGGGCTCTAGTTTATTCCAGTTGTACTTCCT-3′) using Prime STAR GXL DNA Polymerase (Takara), purified with a FastGene Gel/PCR Extraction Kit (Nippon Genetics, Tokyo, Japan), and cloned into a linearized pME18neo-IRIS-ZsGreen1 vector using an In-Fusion HD Cloning Kit (Takara), according to the manufacturers’ instructions. A day before transfection, 293T (5 × 10^5^) or MCF7 (1 × 10^6^) cells were seeded in 6-well plates and incubated at 37 °C in a 5% CO_2_ atmosphere. The next day, the cells were transfected with LXN-plasmid or NC vector using FuGene HD (Promega, Madison, WI, USA), according to the manufacturer’s instructions. The transfection efficiency was evaluated by detecting the number of GFP-expressing cells using an EVOS FL Auto 2 Cell Imaging System (Thermo Fisher Scientific) at 48 h post-transfection and Western blot analysis of cell lysates at 72 h post-transfection.

### 2.8. Flow Cytometry Analysis of Cell Cycle

293T and MCF7 cells transfected with the EFEMP1 siRNA or LXN-plasmid were harvested at 72 h post-transfection and their DNA content was analyzed using flow cytometry, as described previously [59,78]. Briefly, the harvested cells (10^6^) were washed with PBS, fixed in 1% formaldehyde and 70% ethanol, and resuspended in RNase A (Invitrogen; 100 μg/mL) at 37 °C for 20 min. The cells were then stained with propidium iodide (PI; Sigma-Aldrich; 50 μg/mL) for 10 min at room temperature. For each sample, 10,000 events were analyzed using BD Accuri^TM^ C6 Plus with a sampler flow cytometer (Becton-Dickinson, Franklin Lakes, NJ, USA), followed by data analysis using the FlowJo v10 software (FlowJo, LLC, Ashland, OR, USA).

### 2.9. EVOS Fluorescence Microscopy

293T and MCF7 cells transfected with EFEMP1 siRNA or LXN-plasmid were harvested at 48 h post-transfection. The cells (2 × 10^6^) were then seeded in 10 mL plates with DMEM and incubated for an additional 72 h. The growth morphology and proliferation of cells were observed using the trans-light mode of an EVOS FL Auto 2 Cell Imaging System (Thermo Fisher Scientific) as described previously [59].

### 2.10. Statistical Analysis

All data are expressed as the mean ± standard deviation (SD) for at least three independent experiments. The *p*-values were calculated using Student’s *t*-test. Differences between groups were estimated to be significant at *p* < 0.05 (*) and strongly significant at *p* < 0.01 (**) and *p* < 0.001 (***). The statistically analyzed values were plotted using GraphPad Prism version 8 (GraphPad Software, San Diego, CA, USA). For RNA-seq data, statistical analysis was performed using statistical tools integrated in the RaNA-seq web platform, as described previously [60].

## 3. Results

### 3.1. Quality Assessment of RNA-Seq Data

To understand the differences between human cell lines in which BLV induces morphological changes and apoptosis and those in which it does not, we performed a comparative whole-transcriptome RNA-seq analysis of three previously established cell lines stably infected with BLV versus their uninfected controls, after 100 passages. Apoptosis and morphological changes were induced in 293T and MCF7 cells but not in HeLa cells (Table 1) [59]. As is evident from sequencing metrics (Supplementary Table S3), at least 30 million reads per sample were included in this analysis, with more than 75% of the read pairs matched for all samples. Moreover, the mapping rates of reads that were properly matched to the human reference genome GRCh38 ranged from 93.5% to 97.2%. These results confirmed that the sequencing quality was adequate and the data were reliable for subsequent analyses.

In PCA and heatmap clustering analysis (Supplementary Figure S1), BLV-infected cells clustered independently and were distinguishable from uninfected control cells. The consistent gene expression patterns of replicates within each group across the three cell lines validated our data for comparative analysis.

### 3.2. BLV Infection and Latency Altered the Global Transcriptome Profiles of Human Cells

**Figure 1:**
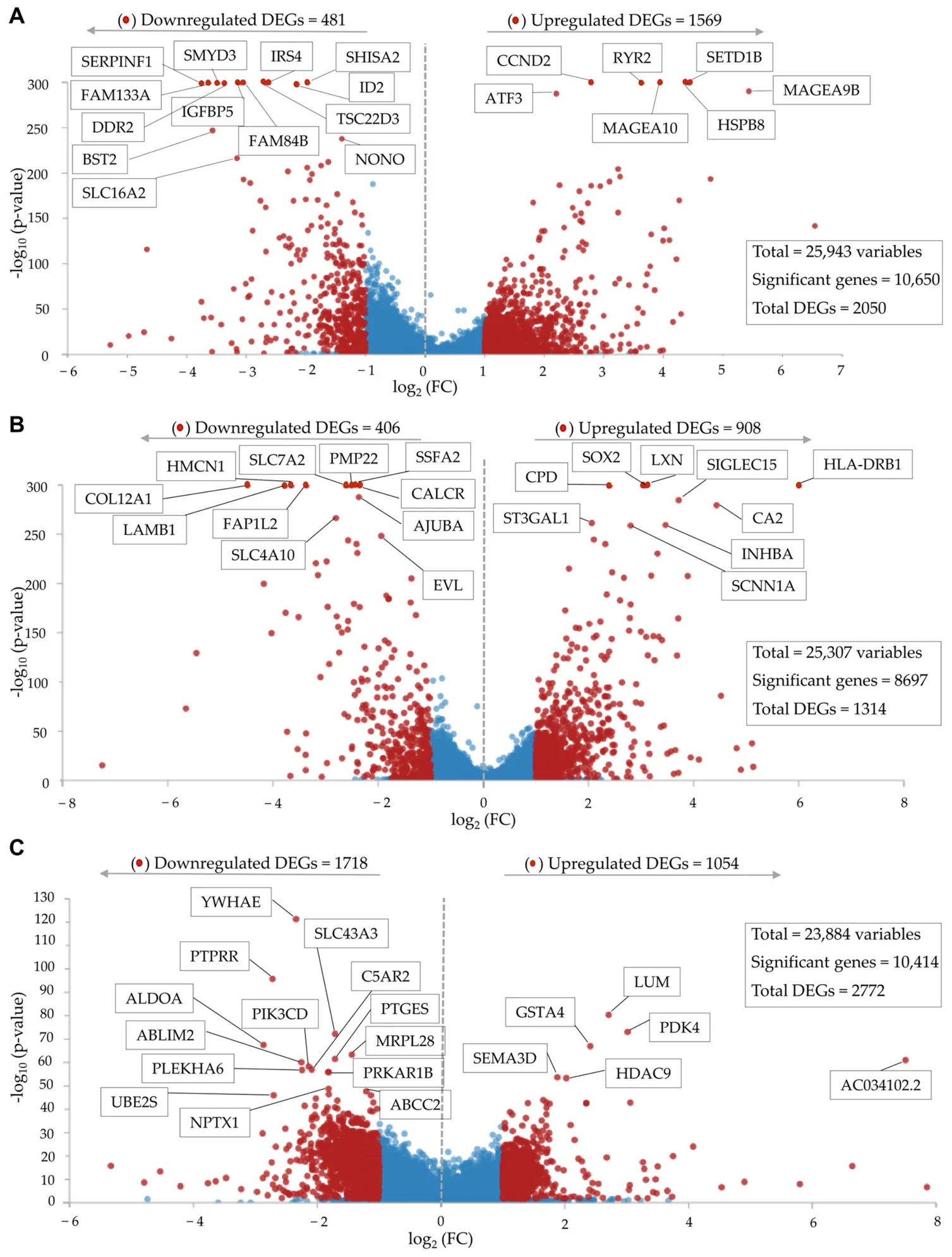
Volcano plots of differentially expressed genes (DEGs) in BLV-infected 293T (**A**), MCF7 (**B**), and HeLa (**C**) vs. control transcriptomes. The vertical dotted gray line corresponds to zero value. Total numbers of analyzed genes, significant genes (*p*-adjust < 0.05), and total DEGs (|log_2_FC| ≥ 1, and *p*-adjust < 0.05) were indicated in a large box on the right side of each plot. The red dots indicate DEGs with |log_2_FC| ≥ 1, and the blue dots indicate those statistically non-significant and/or genes with |log_2_FC| < 1. The number of down- and upregulated DEGs was shown on the upper part of each plot. Gene symbols in small boxes refer to the 20 genes with the lowest *p*-adjust values.

First, we performed a differential gene expression analysis between BLV-infected and uninfected control cells (*n* = 3 for each), for each cell line (Figure 1). In the analysis for the 293T cell line, 25,943 genes were identified, of which 10,650 (41.1%) were statistically significant (*p*-adjust < 0.05) (Figure 1A). Among these significant genes, 2050 DEGs were identified, of which 481 (23.5%) were downregulated and 1569 (76.5%) were upregulated (Figure 1A). The top 20 significant genes among the identified DEGs were stratified into 13 downregulated (*IGFBP5*, *SERPINF1*, *IRS4*, *TSC22D3*, *DDR2*, *FAM84B*, *FAM133A*, *SHISA2*, *SMYD3*, *ID2*, *BST2*, *NONO*, and *SLC16A2*) and 7 upregulated (*CCND2*, *MAGEA10*, *SETD1B*, *HSPB8*, *RYR2*, *MAGEA9B*, and *ATF3*) genes (Figure 1A, Supplementary Table S4). Similarly, in MCF7, among the 25,307 genes identified, 8697 (34.4%) were statistically significant (*p*-adjust < 0.05) (Figure 1B). Among these significant genes, 1314 DEGs were identified, of which 406 (30.9%) were downregulated and 908 (69.1%) were upregulated (Figure 1B). The top 20 significant genes among the identified DEGs were stratified into 11 downregulated (*SLC7A2*, *CALCR*, *LAMB1*, *PMP22*, *COL12A1*, *SSFA2*, *HMCN1*, *AFAP1L2*, *AJUBA*, *SLC4A10*, and *EVL*) and 9 upregulated (*LXN*, *CPD*, *SOX2*, *HLA-DRB1*, *SIGLEC15*, *CA2*, *ST3GAL1*, *INHBA*, and *SCNN1A*) genes (Figure 1B, Supplementary Table S4). Of the 23,884 genes identified in HeLa cells, 10,414 (43.6%) were statistically significant (*p*-adjust < 0.05) (Figure 1C). Among the significant genes, 2772 DEGs were identified, including 1718 (62.0%) downregulated and 1059 (38.0%) upregulated genes (Figure 1C). The top 20 significant genes among the identified DEGs in HeLa cells were stratified into 14 downregulated (*YWHAE*, *PTPRR*, *SLC43A3*, *ALDOA*, *MRPL28*, *PTGES*, *ABLIM2*, *PIK3CD*, *C5AR2*, *PLEKHA6*, *PRKAR1B*, *NPTX1*, *UBE2S*, and *ABCC2*) and 6 upregulated (*LUM*, *PDK4*, *GSTA4*, *AC034102.2*, *SEMA3D*, and *HDAC9*) genes (Figure 1C, Supplementary Table S4).

Overall, the results of the differential expression analysis revealed changes in the transcriptome profiles of the three BLV-infected cells versus the uninfected controls. However, patterns of the mRNA expression of down- (23.5% and 30.9%) and upregulated (76.5% and 69.1%) DEGs were similar in 293T and MCF7 cell lines, respectively. In contrast, the HeLa cell line showed a different pattern, with 62.0% of the DEGs being downregulated and 38.0% being upregulated. Additionally, none of the top 20 significant DEGs were shared between the three cell lines.

### 3.3. Functional Enrichment Analysis Revealed the Involvement of Apoptosis Pathways in BLV-Infected 293T and MCF7 Cell Lines

**Figure 2:**
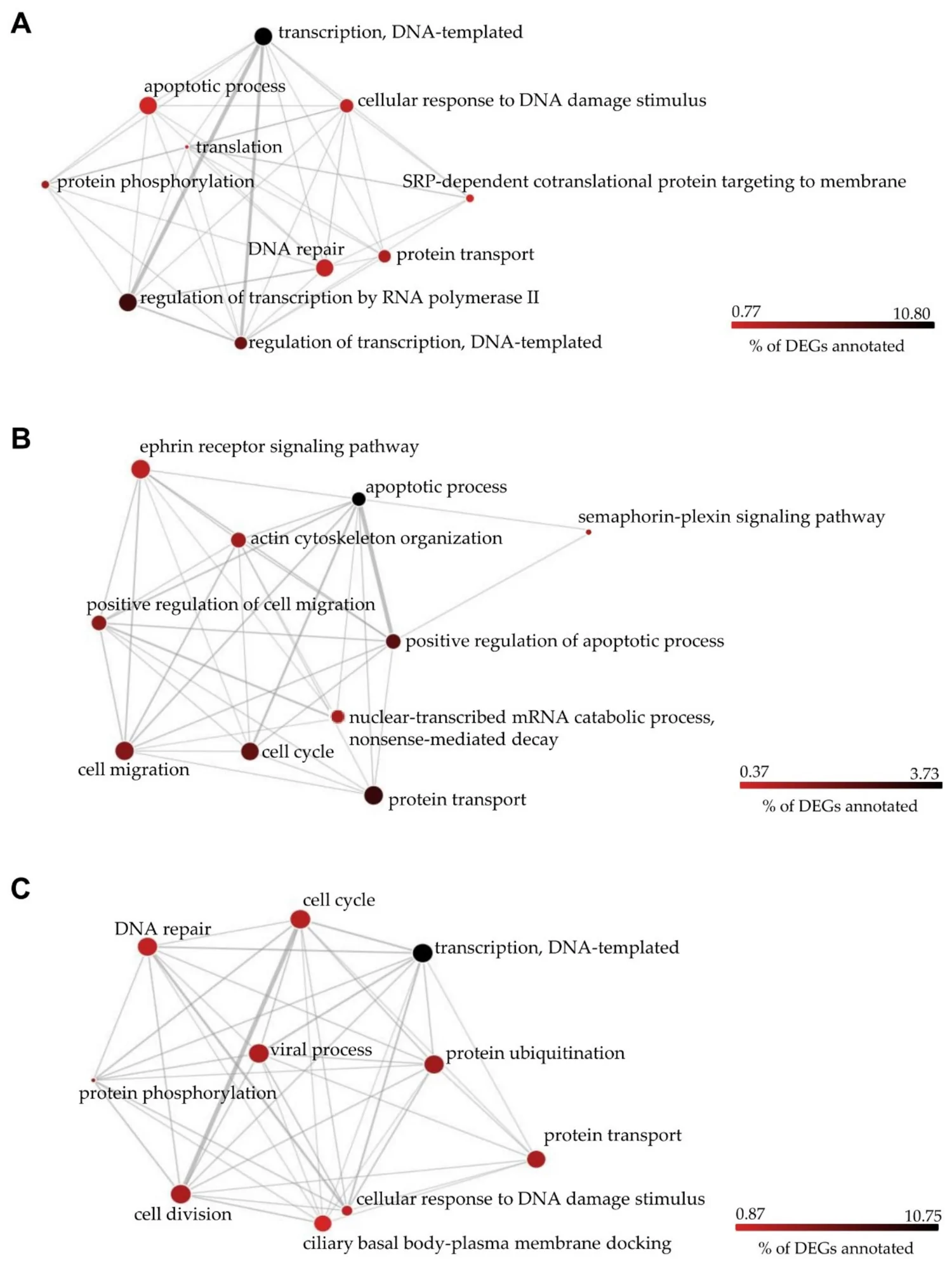
Gene ontology (GO) analysis of biological processes in BLV-infected 293T (**A**), MCF7 (**B**), and HeLa (**C**) cell lines vs. controls. Network of the most significant 10 GO terms of biological processes (*p*-value < 0.05) with the lowest *p*-values shown as indicated beside each node. Each node represents a biological process category. Its color represents the percentage of DEGs annotated to this process, and its size is proportional to the statistical significance of each process.

To obtain a functional perspective of the differences between BLV-infected cell lines and uninfected controls, we performed a functional enrichment analysis of the DEGs. GO enrichment was used to identify processes associated with down- and upregulated genes. In 293T cell line, GO analysis of the top significant 10 biological processes (Figure 2A, Supplementary Table S5) indicated that 10.80% of the DEGs were annotated for “transcription, DNA-templated category,” followed by 7.74% for “regulation of transcription by RNA polymerase II,” and 5.58% for “regulation of transcription, DNA-templated” categories. “protein transport” (2.58%), “DNA repair” (1.60%), “cellular response to DNA damage stimulus” (1.60%), “apoptotic process” (0.95%), and other categories were involved. Similarly, GO analysis of the top significant 10 biological processes in the MCF7 cell line (Figure 2B, Supplementary Table S5) revealed that 3.73% of the DEGs were annotated for “apoptotic process,” followed by 2.84% for “protein transport,” and 2.27% for “positive regulation of apoptotic process” categories. “Cell cycle” (2.14%), “cell migration” (1,63%), “positive regulation of cell migration” (1.49%), and other categories were also involved. For the HeLa cell line, GO analysis of the top significant 10 biological processes (Figure 2C, Supplementary Table S5) showed that 10.75% of the DEGs were annotated for the “transcription, DNA-templated” category, followed by 3.32% for “protein phosphorylation,” and 3.29% for “protein ubiquitination” categories. “Cell division” (2.83%), “protein transport” (2.80%), “viral process” (2.63%), and other categories were involved.

**Figure 3:**
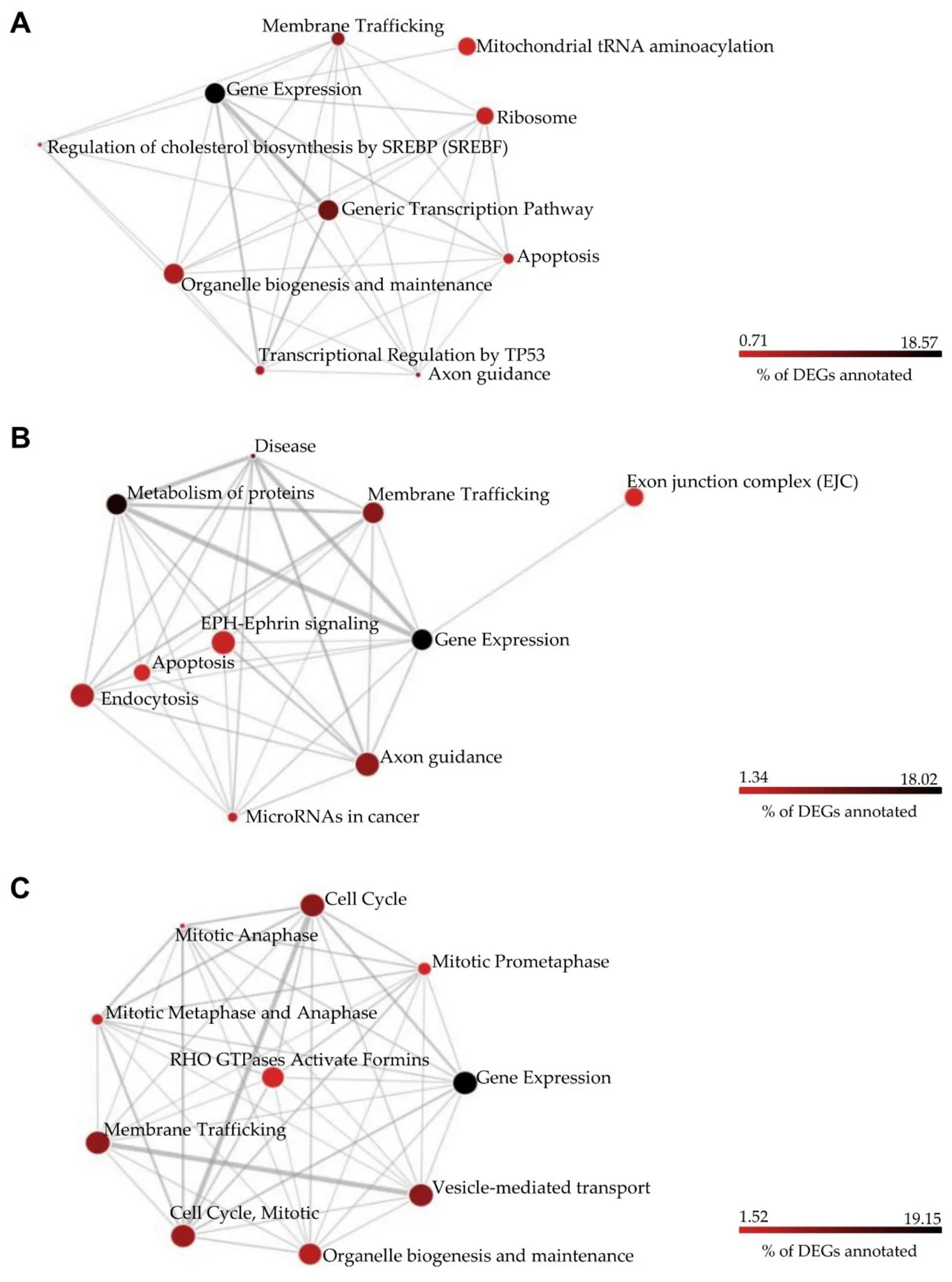
KEGG and REACTOME pathway enrichment analysis in BLV-infected 293T (**A**), MCF7 (**B**), and HeLa (**C**) cell lines vs. controls. The network of the most significant 10 pathways (*p*-value < 0.05) with the lowest *p*-values is shown as indicated beside each node. Each node represents an enrichment pathway. Its color represents the percentage of DEGs annotated to this pathway, and its size is proportional to the statistical significance of each pathway.

The REACTOME and KEGG databases were used to identify the enriched pathways and investigate the molecular interactions involved. In the 293T cell line, the top 10 most significant enriched pathways (Figure 3A, Supplementary Table S6) included 8 REACTOME and 2 KEGG pathways. Among the REACTOME pathways, 18.57% of the DEGs were annotated for “Gene expression,” followed by 8.98% for “Generic Transcription Pathway” and 6.22% for “Membrane Trafficking,” in addition to DEGs annotated for other pathways, whereas 1.70% and 1.13% of the DEGs were annotated for “Ribosome” and “Apoptosis” KEGG pathways, respectively. Similarly, the top 10 significantly enriched pathways in the MCF7 cell line (Figure 3B, Supplementary Table S6) included 6 REACTOME and 4 KEGG pathways. Among the REACTOME pathways, 18.02% of the DEGs were annotated for “Gene expression,” followed by 15.91% for “Metabolism of proteins,” 8,73% for “Disease,” in addition to DEGs annotated for other pathways included, whereas 3.11%, 1.83%, 1.83%, and 1.51% of the DEGs were annotated for “Endocytosis,” “Exon junction complex (EJC),” “MicroRNAs in cancer,” and “Apoptosis” KEGG pathways, respectively. In the HeLa cell line, all top significant 10 enriched pathways were identified in the REACTOME database (Figure 3C, Supplementary Table S6), whereas 19.15% of the DEGs were annotated for “Gene Expression,” followed by 7.35% for “Cell Cycle”, and 7.32% for “Vesicle-mediated transport,” in addition to the DEGs annotated for other pathways involved.

Overall, the results of functional enrichment analysis of DEGs revealed that some GO biological processes and pathways were shared across the three cell lines, whereas “apoptotic process,” “Apoptosis,” and “Axon guidance” pathways were detected in BLV-infected 293T and MCF7 cell lines but not in BLV-infected HeLa cells compared with those in uninfected controls.

### 3.4. Identification of Specific Genes Related to Apoptosis Induced in BLV-Latently Infected Human Cells

To identify specific down- and upregulated genes related to apoptosis, we restricted our analysis to DEGs with |log_2_FC| ≥ 1.5 and *p*-adjust < 0.01 in the three BLV-infected cell lines vs. controls (Table 2). We identified 654, 477, and 577 DEGs in BLV-infected 293T, MCF7, and HeLa cells compared with uninfected controls, respectively (Table 2). Among these DEGs, 163 (24.9%) were downregulated and 491 (75.1%) were upregulated in BLV-infected 293T cell line, whereas 171 (35.8%) were downregulated and 306 (64.2%) were upregulated in BLV-infected MCF7 cell line, and 433 (75.0%) were downregulated and 144 (25.0%) were upregulated in BLV-infected HeLa cell line (Table 2).

**Figure 4:**
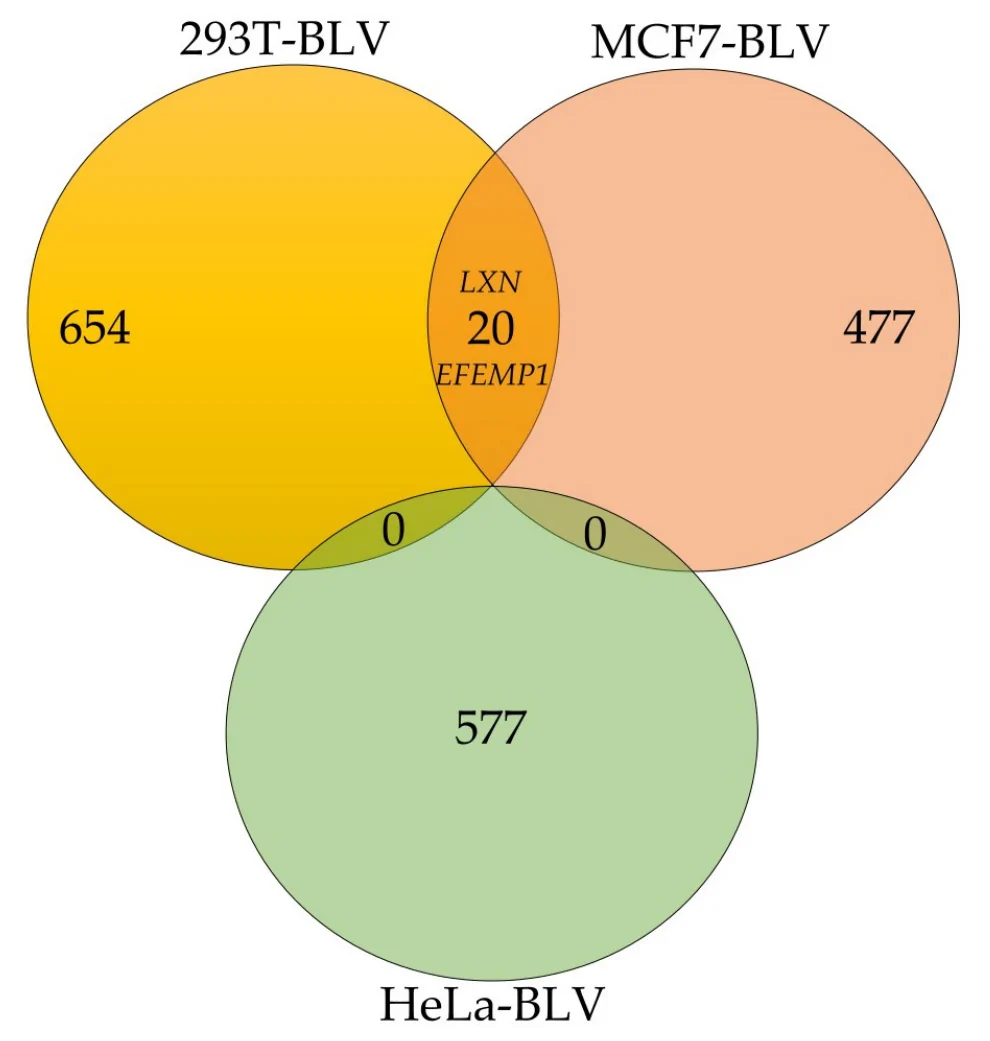
Venn diagram illustrating the number of filtered DEGs (|log_2_FC| ≥ 1.5, and *p*-adjust < 0.01), and the DEGs among them share similar expression in BLV-infected 293T (orange circle), MCF7 (brown circle), and HeLa (green circle) cell lines. The number 20 refers to the DEGs shared between 293T and MCF7 cells, with *EFEMP1* (downregulated) and *LXN* (upregulated) that exhibit |log_2_FC| ≥ 2.5 in both cell lines being selected for further analysis.

When comparing DEGs that displayed similar expression patterns across the three analyzed cell lines, we identified 20 genes that were shared between the 293T and MCF7 cell lines. In contrast, none of the DEGs showed a comparable expression pattern in HeLa cell line analysis (Figure 4).

Among the 20 identified genes (Table 3), six were downregulated (*METTL7A*, *RHOXF1P3*, *NPNT*, *EFEMP1*, *FGFR2*, and *SEMA3D*) and 14 were upregulated (*CA2*, *CYP1A1*, *WNT11*, *LXN*, *TUBA4A*, *LINGO1*, *SLC2A6*, *TCIM*, *DPYSL4*, *SCARA3*, *SHC2*, *PLAC1*, *PDIA2*, and *GALNT14*), with different expression levels in the three cell lines.

**Figure 5:**
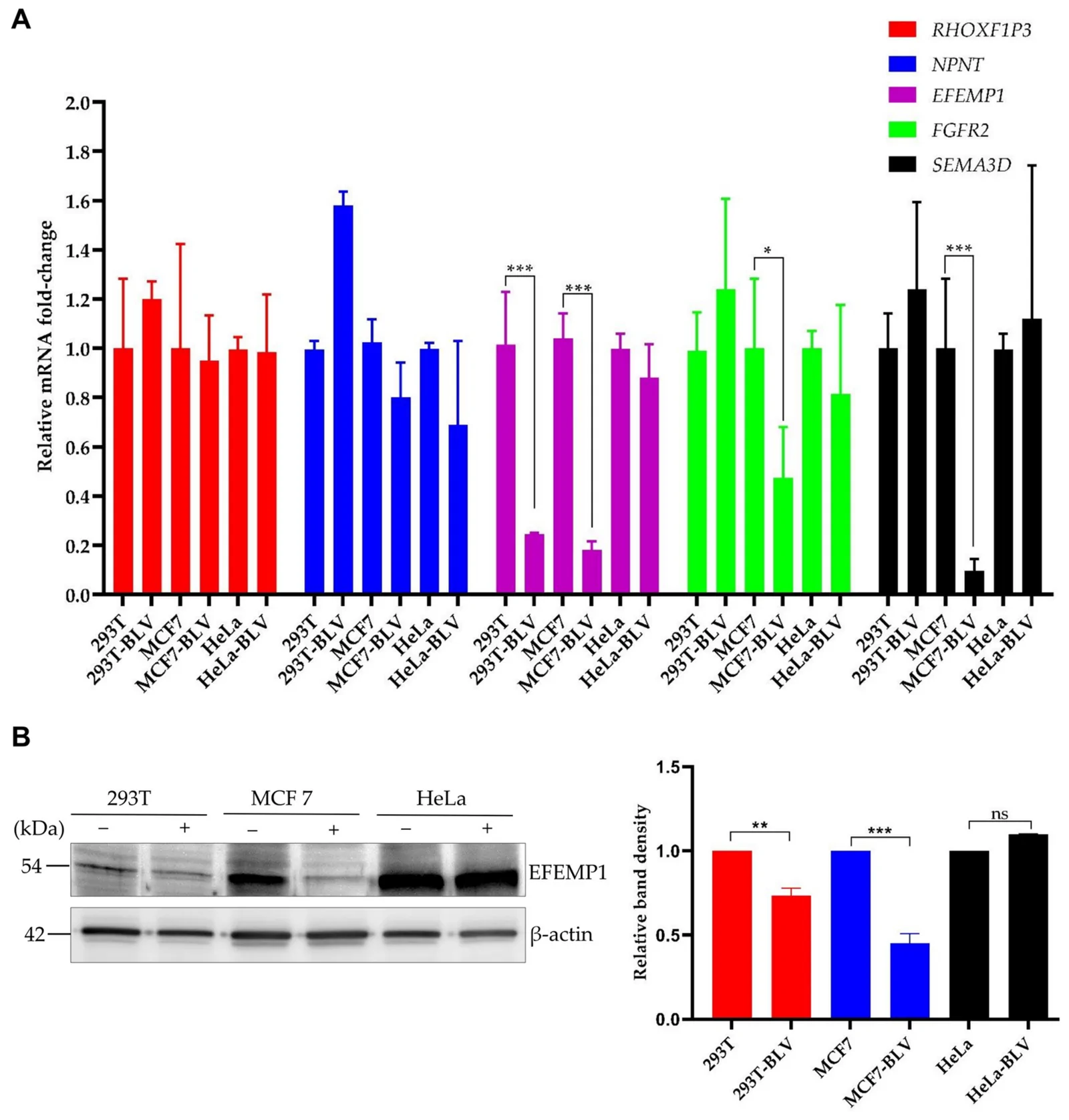
Experimental validation of downregulated genes in BLV-infected 293T, MCF7, and HeLa cell lines vs. controls. (**A**) Using RT-qPCR, mRNA expression levels of 5 downregulated genes selected were measured and normalized to mRNA levels of *GAPDH*. *EFEMP1* gene showed a significant downregulation in BLV-infected 293T and MCF7 cell lines vs. controls, but *FGFR2* and *SEMA3D* genes showed a significant downregulation only in the BLV-infected MCF7 cell line. (**B**) Western blot analysis of EFEMP1 expression levels in cell lysates of controls (−) vs. BLV-infected 293T, MCF7, and HeLa cell lines (+). Cell lysates were prepared, and 30 µg of them were subjected to Western blotting using a rabbit polyclonal EFEMP1/Fibulin-3 antibody (Abcam) or anti-β-actin MAb (Sigma-Aldrich). Positions of the molecular mass markers of both proteins were indicated (left panel). Band densities of EFEMP1 were quantified using ImageJ software (version 1.54p) and normalized to those of β-actin, and the relative band densities in each cell line were shown (right panel). Each column and error bar in A or B represents the mean ± SD for three independent culture experiments. The *p*-values were calculated using a Student’s *t*-test and the asterisk indicates a statistically significant difference (* *p* < 0.05, ** *p* < 0.01, and *** *p* < 0.001), while ns means a non-significant value.

**Figure 6:**
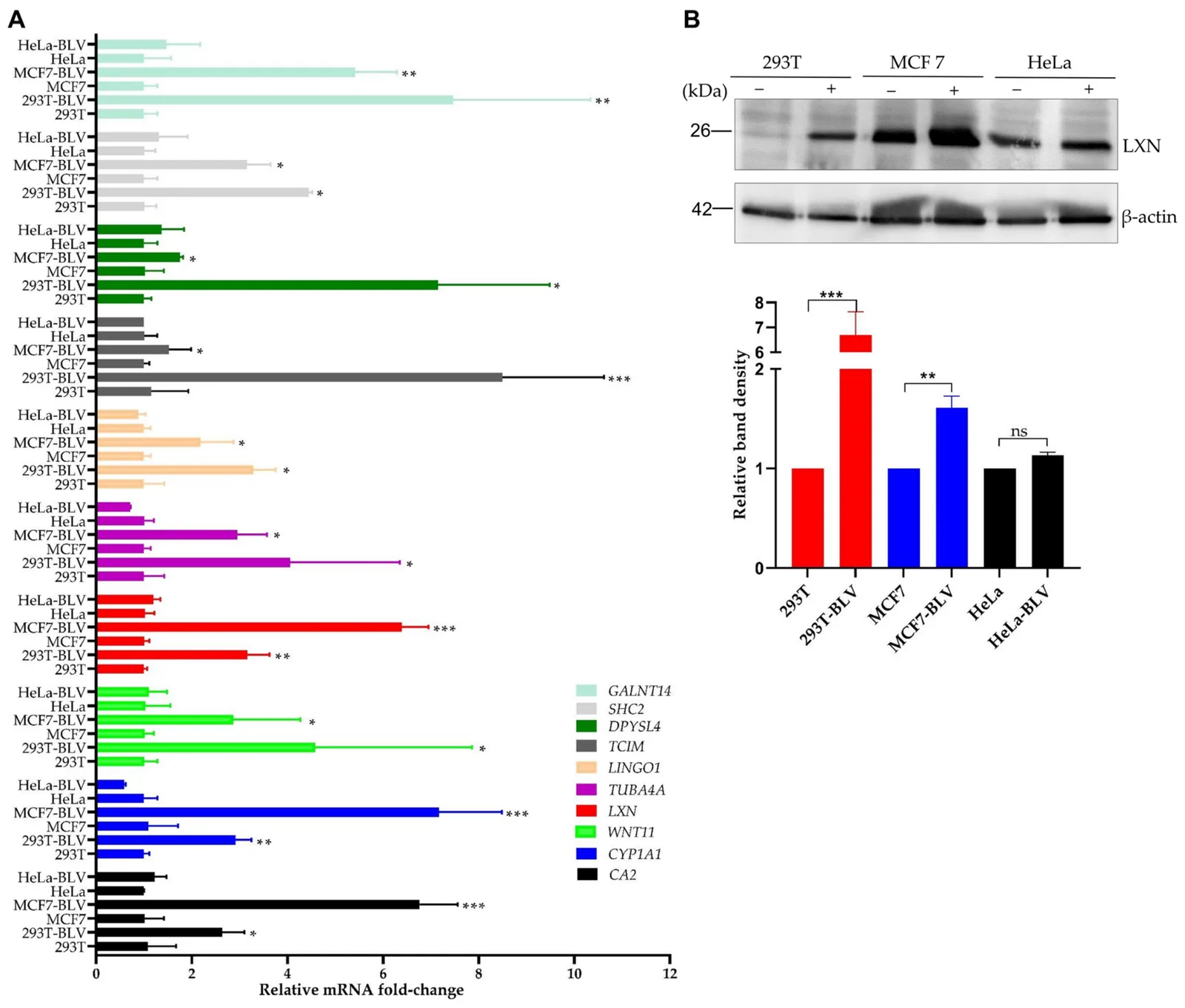
Experimental validation of upregulated genes in BLV-infected 293T, MCF7, and HeLa cell lines vs. controls. (**A**) Using RT-qPCR, mRNA expression levels of 10 upregulated genes selected were measured and normalized to mRNA levels of *GAPDH*. All 10 upregulated genes in this figure showed significant differences in BLV-infected 293T and MCF7 cell lines vs. controls, but not in the BLV-infected HeLa cell line. (**B**) Western blot analysis of LXN expression levels in cell lysates of controls (−) vs. BLV-infected 293T, MCF7, and HeLa cell lines (+). Western blotting of 30 µg of cell lysates using rabbit polyclonal LXN/TCI antibody (Abcam) or anti-β-actin MAb (Sigma-Aldrich) was performed. Positions of the molecular mass markers of both proteins were indicated (**upper panel**). Band densities of LXN were quantified using ImageJ software and normalized to those of β-actin, and the relative band densities in each cell line were shown (**lower panel**). Each column and error bar in A or B represents the mean ± SD for three independent culture experiments. The *p*-values were calculated using a Student’s *t*-test, and the asterisk indicates a statistically significant difference (* *p* < 0.05, ** *p* < 0.01, and *** *p* < 0.001), while ns means a non-significant value.

For validation of the expression levels of the 20 obtained genes, DEGs exhibiting |log_2_FC| ≥ 2.0 in either 293T or MCF7 cell lines were subjected to RT-qPCR. Fifteen genes (5 downregulated and 10 upregulated) were obtained for testing. Among the five downregulated genes (Figure 5A), *EFEMP1* was significantly downregulated in BLV-infected 293T and MCF7 cell lines (*p* < 0.001) vs. controls, whereas *FGFR2* (*p* < 0.05) and *SEMA3D* (*p* < 0.001) were significantly downregulated only in the BLV-infected MCF7 cell line. However, the downregulation of *RHOXF1P3* and *NPNT* could not be confirmed. Additionally, a significant decrease in EFEMP1 protein expression was confirmed in BLV-infected 293T (*p* < 0.01) and MCF7 (*p* < 0.001) cell lines vs. controls using Western blot analysis (Figure 5B). In contrast, the 10 selected upregulated genes (*CA2*, *CYP1A1*, *WNT11*, *LXN*, *TUBA4A*, *LINGO1*, *TCIM*, *DPYSL4*, *SHC2*, and *GALNT14*) showed significant differences in mRNA expression in BLV-infected 293T and MCF7 cell lines vs. controls, but not in the BLV-infected HeLa cell line (Figure 6A). Moreover, a significant increase in LXN protein expression was confirmed in BLV-infected 293T (*p* < 0.001) and MCF7 (*p* < 0.01) cell lines vs. controls using Western blot analysis (Figure 6B).

Overall, the comparative analysis of DEGs enabled us to identify genes with shared expression patterns in BLV-infected 293T and MCF7 cell lines, but not in the BLV-infected HeLa cell line. These genes were expected to be related to apoptosis induced after BLV latency in both cell lines. *EFEMP1* (downregulated) and *LXN* (upregulated) genes that exhibited the highest expression levels in both cell lines (|log_2_FC| ≥ 2.5) were selected for the subsequent knockdown and overexpression experiments, respectively.

### 3.5. EFEMP1 Knockdown Affected the Cell Proliferation, Induced Apoptosis, and Changed the Growth Morphology of Human Cells

**Figure 7:**
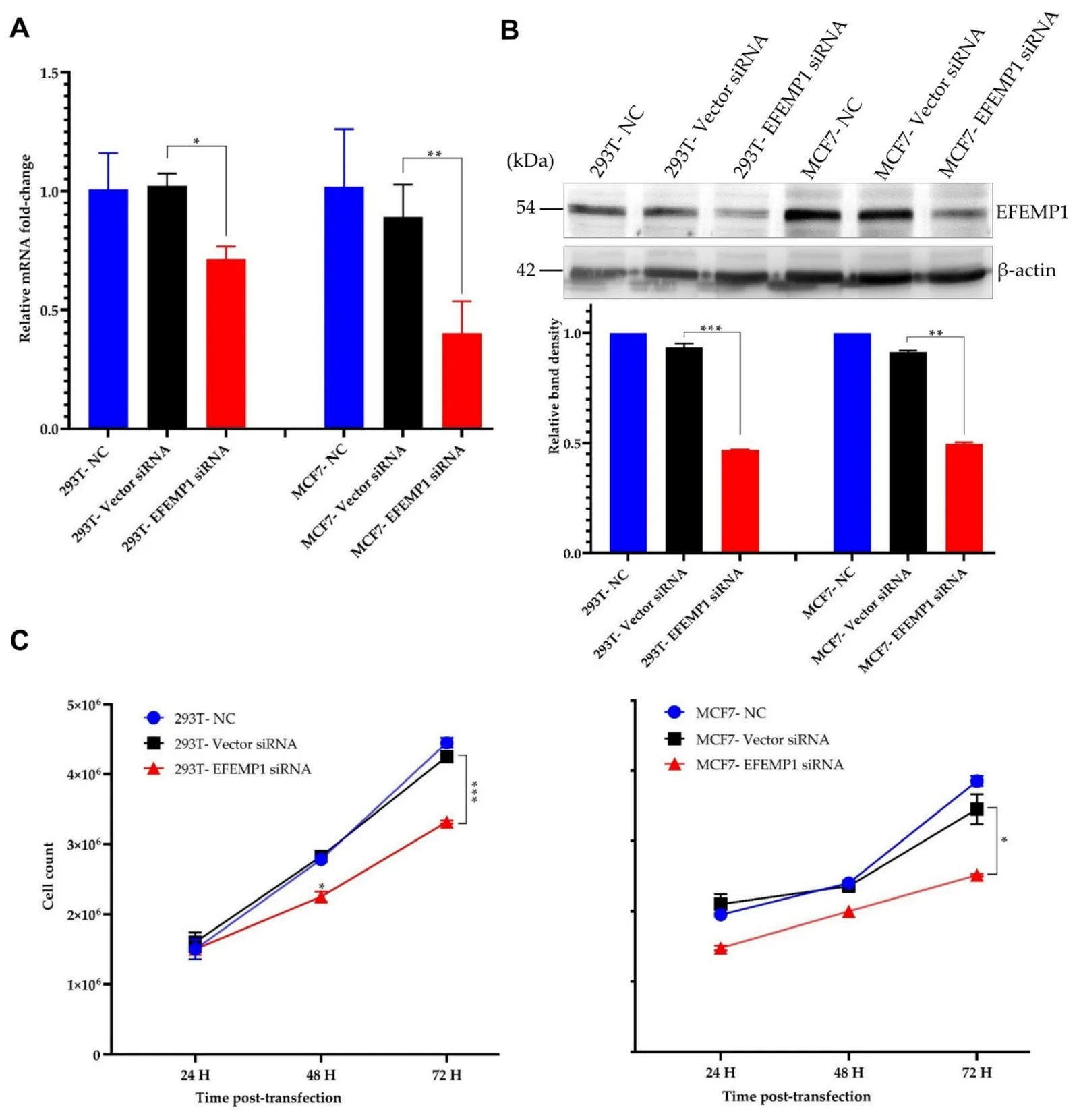

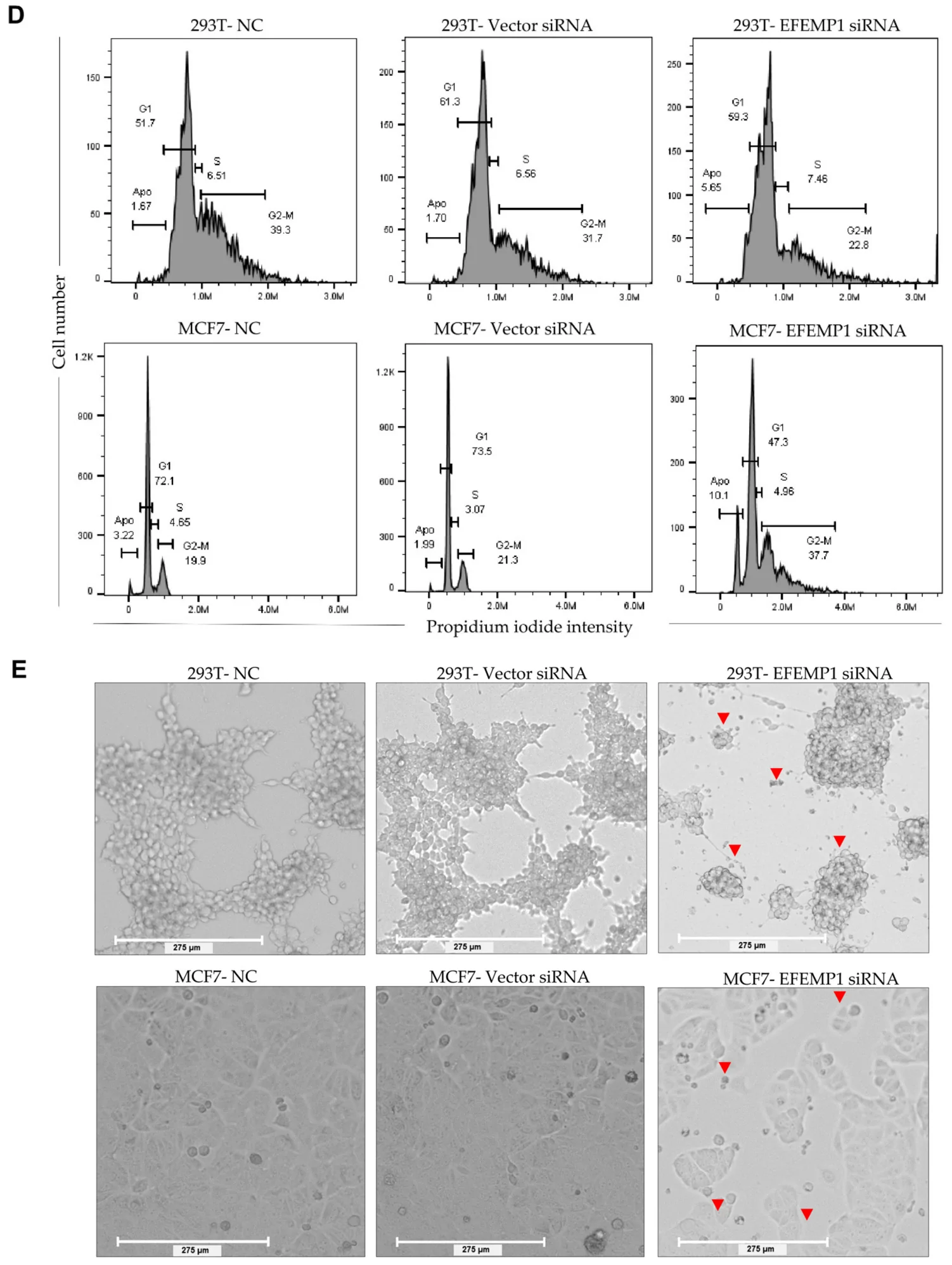
Effect of EFEMP1 knockdown in 293T and MCF7 cell lines. 293T (5 × 10^5^) or MCF7 (1 × 10^6^) cells were seeded in 6-well plates the day prior to transfection with vector siRNA or EFEMP1 siRNA. (**A**) For knockdown efficiency 48 h post-transfection, mRNA expression levels of the *EFEMP1* gene were measured using RT-qPCR and normalized to mRNA levels of *GAPDH*. (**B**) At 72 h post-transfection, EFEMP1 protein expression levels were detected by Western blotting of 30 µg of cell lysates using rabbit polyclonal EFEMP1/Fibulin-3 antibody (Abcam), or anti-β-actin MAb (Sigma-Aldrich). Positions of the molecular mass markers of both proteins were indicated (**upper panel**). Band densities of EFEMP1 were quantified using ImageJ software and normalized to those of β-actin, and the statistical differences in relative band densities between vector siRNA and EFEMP1 siRNA were shown (**lower panel**). (**C**) EFEMP1 knockdown significantly decreased the cell count and proliferation of 293T (**left panel**) and MCF7 (**right panel**) cells, as counted manually every 24 h post-transfection for 72 h after staining with trypan blue. (**D**) EFEMP1 knockdown induced apoptosis in 293T (**upper panel**) and MCF7 (**lower panel**) cells as detected by BD Accuri^TM^ C6 Plus with a sampler flow cytometer (Becton-Dickinson) using PI staining 72 h post-transfection and analyzed by FlowJo v10 software (FlowJo). (**E**) EFEMP1 knockdown changed the growth morphology of 293T (**upper panel**) and MCF7 (**lower panel**) cells. Transfected and negative control cells were harvested 48 h post-transfection, and then 2 × 10^6^ cells were seeded in 10 mL plates and incubated for a further 72 h. Growth morphology and cell proliferation were observed by the trans-light mode of an EVOS FL Auto 2 Cell Imaging System (Thermo Fisher Scientific), using a 10× lens. The red arrows indicate abnormal growth morphology, colonization, fragmented cells, and loss of demarcation of cells. The scale bar, 275 µm, appears in the lower part of each picture. All experiments in this figure were performed in replicates and each column and error bar represents the mean ± SD for three independent transfection experiments. The *p*-values were calculated using a Student’s *t*-test and the asterisk indicates a statistically significant difference (* *p* < 0.05, ** *p* < 0.01, and *** *p* < 0.001).

RT-qPCR (Figure 7A) and Western blot (Figure 7B) analyses showed that the mRNA expression and protein levels of EFEMP1 were significantly decreased in 293T (*p* < 0.001) and MCF7 (*p* < 0.01) cells transfected by the EFEMP1 siRNA, respectively. Cell count analysis indicated that EFEMP1 siRNA significantly decreased the proliferation of 293T transfected cells after 48 h (*p* < 0.05) and strongly decreased it after 72 h (*p* < 0.001) compared with that of vector siRNA-transfected cells (Figure 7C, left panel). Similarly, EFEMP1 siRNA transfection significantly decreased the cell count of MCF7 cells after 72 h (*p* < 0.05) compared with that of vector siRNA-transfected cells (Figure 7C, right panel). The number of apoptotic 293T and MCF7 cells and cells in different stages of the cell cycle after EFEMP1 siRNA transfection were compared with those of cells transfected with vector siRNA using PI staining flow cytometry (Figure 7D). In 293T cells, EFEMP1 siRNA induced apoptosis in 5.56% of cells, with a decreased number of cells being in the G2/M phase (22.8%), compared with an apoptotic rate of 1.70% in vector siRNA-transfected 293T cells, with 31.7% of the cells being in the G2/M phase. A higher apoptotic rate (10.1%) and number of cells in the G2/M phase (37.7%), with a decreased number of cells in G1 phase (47.3%), were observed in MCF7 cells transfected by EFEMP1 siRNA, compared with 1.99% apoptotic rate, 21.3% cells in the G2/M phase, and 73.5% cells in the G1 phase for MCF7 cells transfected with vector siRNA. Growth morphology of 293T and MCF7 cells was obviously changed after transfection with EFEMP1 siRNA compared with that of vector siRNA-transfected and control cells (Figure 7E). Cell migration was obviously decreased in EFEMP1 siRNA-transfected 293T and MCF7 cells, with abnormal changes in growth morphology, including colonization, fragmented cells, and loss of demarcation of cells, unlike the confluent and normal culture morphology of both cell lines in the control groups.

### 3.6. LXN Overexpression Affected Cell Proliferation, Induced Apoptosis, and Changed the Growth Morphology of Human Cells

**Figure 8:**
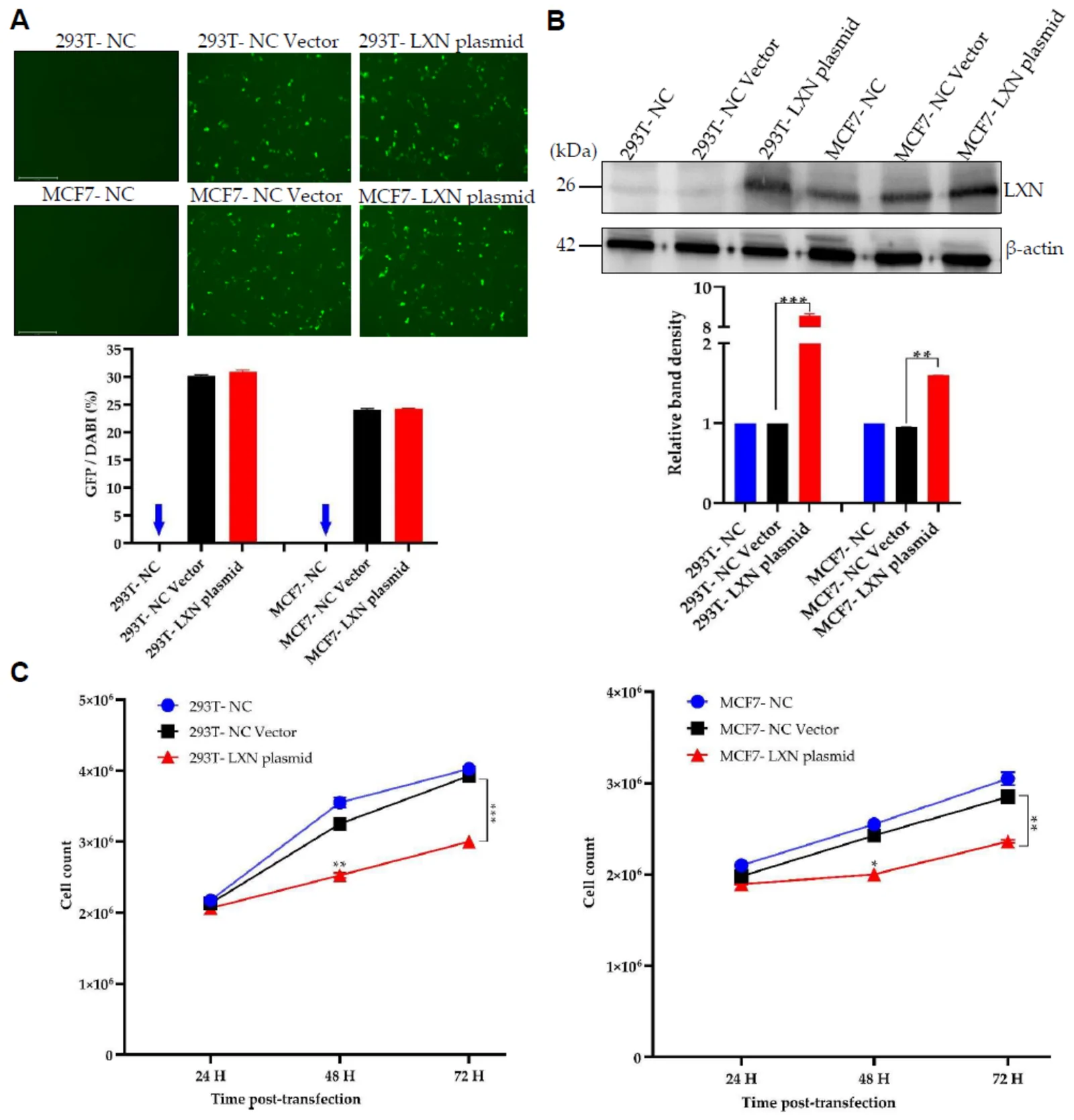

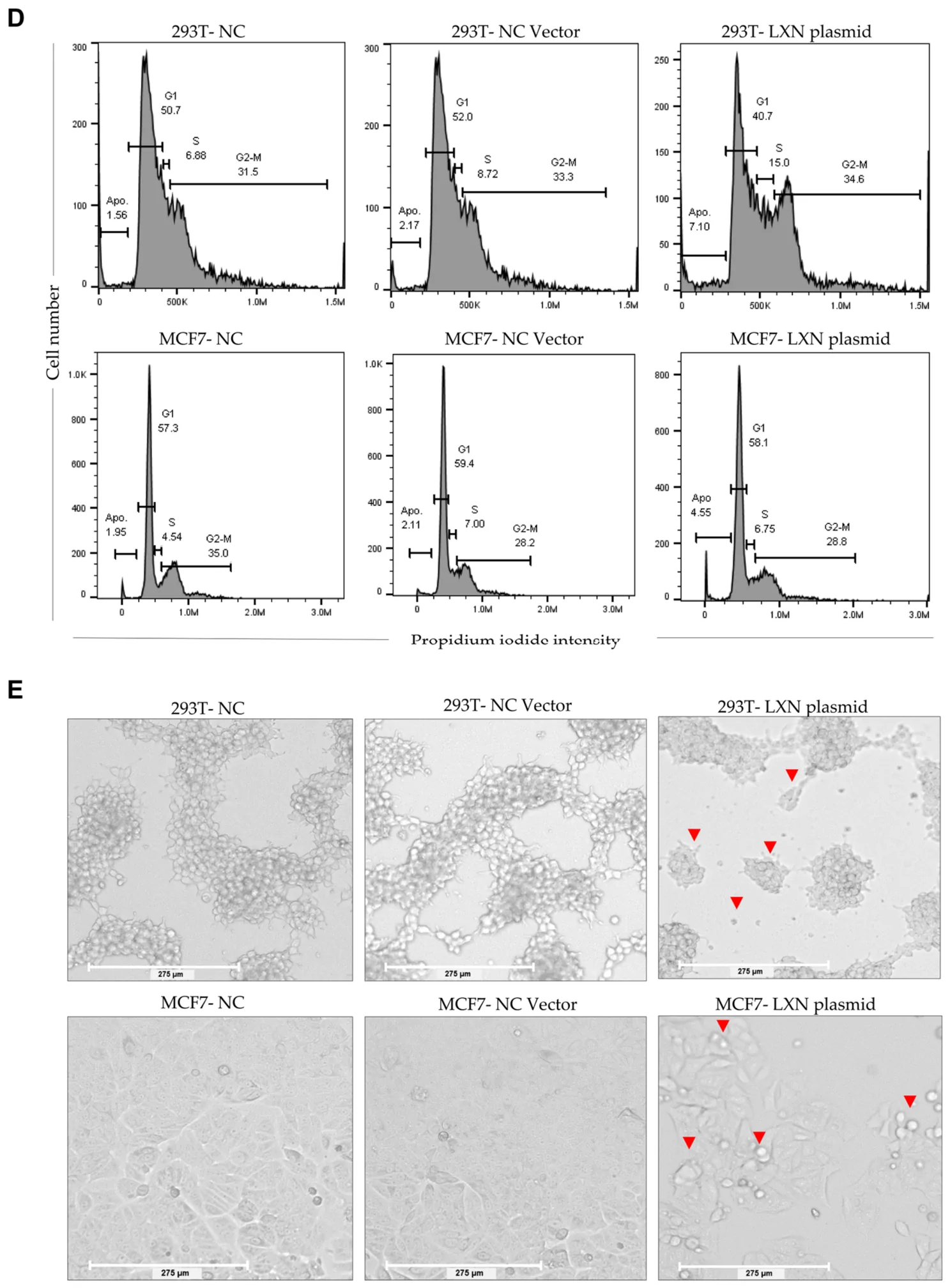
Effect of LXN overexpression in 293T and MCF7 cell lines. 293T (5 × 10^5^) or MCF7 (1 × 10^6^) cells were seeded in 6-well plates the day prior to transfection with 4 µg of LXN-plasmid or NC vector. (**A**) For transfection efficiency 48 h post-transfection, GFP expression in transfected cells was observed by an EVOS FL Auto 2 Cell Imaging System (Thermo Fisher Scientific), using a 10× lens (**upper panel**). Nuclei were stained with 1/2000 medium containing 10 μg/mL Hoechst 33342 (DAPI; Sigma-Aldrich), and then the merged images of overlaid Hoechst and GFP were scanned and counted computationally. The transfection efficiency was expressed as the percentage of GFP-expressing cells relative to DABI (**lower panel**). Blue arrows refer to the zero value of non-transfected cells. (**B**) After 72 h, LXN protein expression levels were detected by Western blotting of 30 µg of cell lysates using rabbit polyclonal LXN/TCI antibody (Abcam) or anti-β-actin MAb (Sigma-Aldrich). Positions of the molecular mass markers of both proteins were indicated (**upper panel**). Band densities of LXN were quantified using ImageJ software and normalized to those of β-actin, and the statistical differences in relative band densities between the NC vector and LXN-plasmid were shown (**lower panel**). (**C**) LXN overexpression significantly decreased the cell count and proliferation of 293T (**left panel**) and MCF7 (**right panel**) cells, as counted manually every 24 h post-transfection for 72 h after staining with trypan blue. (**D**) LXN overexpression induced apoptosis in 293T (**upper panel**) and MCF7 (**lower panel**) cells, as detected by BD Accuri^TM^ C6 Plus with a sampler flow cytometer (Becton-Dickinson) using PI staining 72 h post-transfection and analyzed by FlowJo v10 software (FlowJo). (**E**) LXN overexpression changed the growth morphology of 293T (**upper panel**) and MCF7 (**lower panel**) cells. Transfected and negative control cells were harvested 48 h post-transfection, and then 2 × 10^6^ cells were seeded in 10 mL plates and incubated for a further 72 h. Growth morphology and cell proliferation were observed by the trans-light mode of an EVOS FL Auto 2 Cell Imaging System (Thermo Fisher Scientific), using a 10× lens. The red arrows indicate abnormal growth morphology, colonization, fragmented cells, and loss of demarcation of cells. The scale bar, 275 µm, appears in the lower part of each picture. All experiments in this figure were performed in replicates and each column and error bar represents the mean ± SD for three independent transfection experiments. The *p*-values were calculated using a Student’s *t*-test, and the asterisk indicates a statistically significant difference (* *p* < 0.05, ** *p* < 0.01, and *** *p* < 0.001).

GFP expression (Figure 8A) revealed that 30.1% and 31.2% of 293T cells, and 24.9% and 25.1% of MCF7 cells were successfully transfected by the NC vector or LXN-plasmid, respectively. Additionally, Western blot analysis (Figure 8B) showed significantly higher levels of LXN protein in 293T (*p* < 0.001) and MCF7 (*p* < 0.01) cells transfected with LXN-plasmid than the levels in the same cells transfected with the NC vector. LXN-plasmid–transfected 293T cells exhibited a significant decrease in cell count after 48 h (*p* < 0.01) and a strong decrease after 72 h (*p* < 0.001) compared with the counts of cells transfected with the NC vector (Figure 8C, left panel). Similarly, a decrease in cell count was found in LXN-plasmid–transfected MCF7 cells after 48 h (*p* < 0.05) and 72 h (*p* < 0.01) compared with the counts of MCF7 cells transfected with the NC vector (Figure 8C, right panel). The apoptotic rates and changes in DNA content of 293T and MCF7 cells transfected with LXN-plasmid are shown in Figure 8D. Transfection of 293T cells with LXN-plasmid resulted in an apoptotic rate of 7.10%, with decreased numbers of cells in the G1 (40.7%) and arrested S (15.0%) phases compared with an apoptotic rate of 2.17% in NC vector-transfected 293T cells, with 52.0% and 8.72% of the cells in the G1 and S phases, respectively. An apoptotic rate of 4.55% was noted in LXN-plasmid–transfected MCF7 cells compared with 2.11% in NC vector-transfected MCF7 cells. As shown in Figure 8D, LXN-plasmid transfection changed the growth morphology of 293T and MCF7 cells compared with that of NC vector-transfected cells. Decreased cell migration and abnormal morphology of the cell culture, such as colonization, fragmented cells, and loss of demarcation of cells, were observed in LXN-plasmid–transfected 293T and MCF7 cells, but not in the NC vector-transfected 293T and MCF7 cells or in the non-transfected control cells.

## 4. Discussion

To understand the behavior of BLV in human cells, we performed comparative whole-transcriptome analysis on single cells from three human cell line models (293T, MCF7, and HeLa), stably infected with BLV, and uninfected controls. Based on our findings, we arrived at three main conclusions. First, the three BLV-infected human cells exhibited differences in their transcriptome profiles, with contrasting patterns of differential gene expression, suggesting that BLV engages different mechanisms in different human cells. Second, the GO functional enrichment analysis revealed that “protein transport” GO process, “Gene expression,” and “Membrane trafficking” pathways were targeted across the three cell line, but “Apoptosis” and “Axon guidance” pathways were exclusively enriched in 293T and MCF7 cell lines, supporting our previous findings of BLV latency-induced apoptosis in 293T and MCF7 cell lines, but not in HeLa cell line [59]. Unlike 293T and MCF7 cells, BLV-infected HeLa cells displayed no cytopathic changes or apoptotic induction, maintaining cellular homeostasis with negligible impact on apoptosis pathways. This outcome is consistent with the lineage of HeLa cells, which originated from a highly aggressive cervical carcinoma known for its robust intrinsic resistance to apoptosis [59]. A previous microarray-based gene expression analysis showed that Tax, a transcriptional activator of BLV, exhibits expression as a stress response in addition to roles in host cell transcription, signaling, and immune responses in HeLa cells [50]. Specifically, BLV Tax regulates heat-shock proteins (HSPs), such as DNAJB1, HSPA1A, and HSPA6, and the JNK and p38 MAPK signaling pathways, thereby inhibiting apoptosis in HeLa cells [50]. Moreover, BLV may contribute to cancer via unknown mechanisms, distinct from those in infected cattle [58]. Third, the comparative analyses of DEGs obtained from the three cell lines enabled us to detect some genes with an expected link to apoptosis induced in latently infected human cells. Among these genes, *EFEMP1*-downregulated and *LXN*-upregulated genes were confirmed to inhibit cell proliferation, alter the growth morphology, and induce apoptosis in 293T and MCF7 cell lines, supporting the notion that they are tumor-suppressor genes.

To our knowledge, the impact of BLV infection on the human cell transcriptome has not been investigated; however, the transcriptome of bovine MAC-T cells stably infected with BLV was analyzed in one study using RNA-seq [56]. In contrast, the transcriptomes of PBMCs and lymphoid tissues from BLV-infected cows have been thoroughly investigated [52,53,54,55]. We identified 2050, 1314, and 2772 DEGs in the analysis of 293T, MCF7, and HeLa cell lines, respectively, at a cut-off value of |log_2_FC| ≥ 1 and *p*-adjust < 0.05. This screening threshold is widely established across multiple RNA-seq studies investigating BLV infection in animal models and cell lines [53,54,56]. These counts are comparable to those of DEGs identified in the transcriptomes of cattle infected with BLV vs. uninfected cows [52,53,54,55]. Nishimori et al. (2023) detected 958 DEGs in the BLV-infected vs. healthy cattle comparison [52], whereas Peterson et al. (2025) reported 2216 DEGs in the comparative transcriptome analysis of cows infected with HPVL vs. LPVL [55]. In contrast, Cuesta et al. (2020) reported a lower number of DEGs, identifying only 352 DEGs in the comparison of the transcriptomes of BLV-infected MAC-T and uninfected cells [56]. Notably, we observed similar mRNA expression patterns in 293T and MCF7 cells, with the majority (76.5% and 69.1%) of identified DEGs being upregulated. In contrast, the transcriptome profiling of the HeLa cell line showed that the majority (62.0%) of identified DEGs were downregulated. Notably, the mRNA expression profiles of 293T and MCF7 cells were in agreement with those reported in several previous studies that showed alterations in the transcriptomes of BLV-infected PBMCs, tissues, or bovine cells versus the controls [52,54,55,56]. For example, a GO analysis of 607 of 958 identified DEGs between the BLV-infected cattle and uninfected group revealed the upregulation of several pathways involved in cellular metabolic processes, cell cycle, biosynthetic process, chromosome organization, cellular response to DNA damage stimulus, and DNA repair [52], whereas González-Méndez et al. [54] reported that *MHC class II*, transcription activation factors, and anti-inflammatory cytokine pathways were upregulated in persistent lymphocytosis cattle compared with an uninfected group. Additionally, Peterson et al. [55] reported that out of 1908 genes with FC ≥ |1.5|, 774 were downregulated and 1134 upregulated genes in cows infected with HPVL vs. LPVL, whereas immune response pathways were downregulated and processes related to cell cycle regulation, mitotic division, and DNA biosynthesis were enriched [55]. Moreover, Cuesta et al. [56] reported that 211 of 352 DEGs were upregulated in BLV-infected MAC-T vs. uninfected cells, with enrichment for pathways involved in the defense response to viruses and collagen catabolism as well as some protooncogenes and tumor suppressor genes. In contrast, Scull et al. (2024) reported that the mRNA expression profiles of BLV-infected cattle were similar to those of the BLV-infected HeLa cell line; among 18,011 genes identified, 571 were upregulated and 1484 were downregulated in BLV+ vs. BLV−PBMCs, with enrichment for a wide variety of immunological pathways under BLV infection [53]. Using microarray analysis, our group previously showed a different mRNA expression pattern in HeLa cells after induction of BLV-Tax protein expression, with the majority of DEGs being upregulated [50]. The different characteristics of infected cells may influence this variability [79]. These findings are also supported by the hypothesis that BLV engages different mechanisms in human cancers [58].

Suppressed or activated enrichment of GO processes and pathways identified in functional analyses is mediated by the proportion of down- and upregulated DEGs annotated in each category [53,56,60]. We focused on the most significant 10 GO terms for biological processes and pathways across the three cell lines analyzed, as described previously [51,56]. Although BLV remained silent in all three human cell lines [59], the latent BLV infection significantly altered several host regulatory pathways, with the “protein transport” GO process, “Gene expression”, and “Membrane trafficking” pathways emerging as the most enriched GO categories across the three human cell lines. The enrichment of “protein transport” genes aligns with the notion of dependence of retroviruses on host intracellular trafficking pathways to maintain viral persistence even with a minimal viral transcription [80,81]. Alterations in “Gene expression” pathways agree with previous findings that BLV latency can modify host transcriptional programs through its regulatory elements and associated epigenetic alterations, allowing infected cells to persist even in the absence of detectable expression of viral genes [1,82,83]. Similarly, changes in genes of the “Membrane trafficking” pathways reflect the needs of retroviruses for vesicular transport and membrane remodeling during assembly, budding, and maintenance of latency [77,81,84]. Several other enriched GO processes and pathways were unique to each cell line, supporting the hypothesis that BLV follows different mechanisms in human cells [58]. For example, “Cell Cycle”, “DNA repair”, and “protein phosphorylation” terms were enriched. These GO processes were also reported to be enriched in the comparison of blood from BLV-infected cows or BLV-infected bovine cell lines versus controls [50,51,52]. In contrast to our findings regarding the absence of enriched immunological pathways, several RNA-seq studies have revealed the enrichment of host immunological pathways in BLV-infected cows or cell lines [53,54,56]. Consistent with and further confirming our previous findings that BLV induces apoptosis and morphological changes after latency in 293T and MCF7 cell lines [59], “Apoptosis” and “Axon guidance” pathways were significantly enriched in the 293T and MCF7 cell lines, but not in the HeLa cell line. The mechanism of apoptosis induced in 293T and MCF7 cell lines, including the caspase cascade pathway and the Bcl-2 family involved, was thoroughly investigated in our previous study [59]. Moreover, our earlier studies showed that apoptosis induced in 293T cells may be triggered by the Tax protein of BLV [21,50]. However, the “Axon guidance” pathway comprises signaling cues that direct cell movement by regulating cytoskeletal dynamics, particularly within the growth cone [85], influencing cell migration and cytoskeletal remodeling [86].

We identified 20 genes, with expected links to apoptosis and morphological changes, through the comparative analysis of DEGs that exhibited |log_2_FC| ≥ 1.5 across the three cell lines [55]. This threshold was also used in multiple RNA-seq studies investigating BLV infection to filter specific upregulated and downregulated genes for validation [53,55,56]. Notably, none of these genes was listed as a human cancer-related gene or even related to BLV integration in cattle [87]. Among these genes, we selected those with |log_2_FC| ≥ 2.0 in either cell line for validation via RT-qPCR [52,55]. Subsequently, the validated genes found to have a similar expression pattern, with |log_2_FC| ≥ 2.5, in 293T and MCF7 cell lines were considered of high importance for apoptosis and morphological changes. Under these criteria, *EFEMP1* and *LXN* genes were selected. Our results confirmed the importance of both genes, as EFEMP1 knockdown or LXN overexpression inhibited cell proliferation, induced apoptosis, and altered the growth morphology of 293T and MCF7 cells. However, the mechanisms by which the BLV targeted these genes in human cells remain unknown.

Our findings are consistent with those of several studies wherein the tumor-suppressive role of *EFEMP1* was documented across multiple cancer types [88,89,90,91]. *EFEMP1* also contributes to tissue structural integrity, which is indicative of its broader biological importance [68]. In gliomas, *EFEMP1* suppresses malignancy by modulating the extracellular microenvironment [88,91]. *EFEMP1* knockdown suppressed the proliferation, migration, and invasion of cells, and promoted apoptosis in glioblastoma cells [91]. Similarly, in gastric cancer and endometrial carcinoma, *EFEMP1* was reported to inhibit proliferation, migration, and in vivo tumor growth, which confirms its tumor-suppressive role [89,90].

The *LXN* gene has also been reported to perform tumor-suppressive roles in several types of cancer; it is often downregulated or silenced in malignancy, and its loss is associated with increased proliferation, invasion, and tumor progression [92,93,94,95]. For example, in human gastric carcinomas, LXN expression is markedly reduced, and molecular evidence suggests that upregulation of *LXN* suppresses cell growth and induces apoptosis, confirming its antitumor activity [92]. Similarly, LXN is downregulated in approximately 50% of melanomas, and exogenous expression of LXN in melanoma cell lines significantly inhibited tumor cell proliferation [93]. In hepatocellular carcinoma, LXN downregulation promoted tumor progression, whereas its re-expression significantly reduced proliferation and colony formation [94]. LNX is downregulated in prostate cancer, whereas its overexpression in prostate luminal cells exhibits significant indirect effects on retinoid metabolism and IFN-associated inflammatory responses [95].

Taken together, our findings are consistent with previous evidence showing that *EFEMP1* and *LXN* act as key regulators of cancer progression, and that opposing alterations in their expression can drive apoptosis and induce notable morphological changes in human cells. Further investigations are required to clarify how BLV targets these two genes to induce apoptosis and morphological alterations in human cells, and to demonstrate the roles of other apoptosis-related genes identified in this study.

Despite the importance of our findings, several limitations should be acknowledged. In particular, we did not investigate the specific molecular mechanisms by which BLV targets and modulates *EFEMP1* and *LXN,* nor their levels during different stages of BLV infection. Additionally, functional knockdown and overexpression assays were conducted in uninfected parental cells instead of BLV-infected clones because the BLV-infected 293T and MCF7 cells showed colonization, easy detachment, and a high number of apoptotic cells [59], making phenotypic reversal experiments very difficult. Therefore, phenotypic recovery experiments in BLV-infected cells and further apoptotic marker assays after knockdown and overexpression will be priorities for our future research. Another limitation of this study is that functional assays were restricted to *EFEMP1* and *LXN* among the 20 candidate genes. The remaining genes represent important targets for our current and future investigations. This study enhances our understanding of the behavior, biological mechanisms, and pathological features of BLV infection in human cells and helps resolve the controversy regarding its potential as a zoonotic pathogen. To our knowledge, this study represents the first whole-transcriptome analysis of human cell lines with stable BLV infection and latency, employing RNA-seq technology. Our results reveal notable variability in the global transcriptomic profiles, as well as in the biological processes and pathways targeted by BLV across the three human cell lines. In addition, we identified several genes linked to apoptosis and morphological changes observed in human cells during BLV latency. The identified genes may be investigated for their potential roles in other virus-induced diseases in humans, including those caused by HIV and HTLV-1. Although these findings provide important insights, future research is needed to elucidate the specific roles of these genes in BLV-induced apoptosis and cellular changes and the mechanisms involved therein.

## Figures and Tables

**Table 1:** Characteristics of BLV-stably infected human cell lines subjected to RNA-seq.

Single Clone	BLV Existence ^a^	BLV Silencing ^b^	Apoptosis	Morphological Changes
293T-BLV	+	+	+	++
MCF7-BLV	+	+	++	+
HeLa-BLV	+	+	−	−

^a^ Detected by BLV full-length PCR (≃8 kb), partial *env* gene nested PCR, or CoCoMo-qPCR targeting LTRs. ^b^ No cellular expression of viral gp51 and p24 proteins, cell-to-cell infectivity, or detectable virion p24 and RNA.

**Table 2:** The number of filtered DEGs obtained from the three analyzed human cell lines.

Single Clone	Total No. of DEGs ^a^	Total No. of Filtered DEGs ^b^	No. of Filtered Downregulated DEGs (%)	No. of Filtered Upregulated DEGs (%)
293T-BLV	2050	654	163 (24.9)	491(75.1)
MCF7-BLV	1314	477	171 (35.8)	306 (64.2)
HeLa -BLV	2772	577	433 (75.0)	144 (25.0)

^a^ DEGs with |log_2_FC| ≥ 1, and *p*-adj < 0.05. ^b^ DEGs with |log_2_FC| ≥ 1.5, and *p*-adj < 0.01.

**Table 3:** Expression levels of DEGs shared similar expression patterns in BLV-infected 293T and MCF7 cell lines compared with their expression in the HeLa cell line analysis.

Gene			293T-BLV ^a^		MCF7-BLV ^a^		HeLa-BLV ^b^	
Downregulated (6)	**Symbol**	**Name**	**log_2_FC**	***p*** **-adj**	**log_2_FC**	***p*** **-adj**	**log_2_FC**	***p*** **-adj**
	*METTL7A*	Methyl transferase-like protein 7A	−1.606	1.2 × 10^−72^	−1.667	2.3 × 10^−29^	0.467	6.90 × 10^−12^
	*RHOXF1P3*	Rhox Homeobox Family Member 1 Pseudogene 3	−1.652	1.0 × 10^−14^	−2.103	4.0 × 10^−10^	0.305	0.077
	*NPNT*	Nephronectin	−1.515	1.3 × 10^−67^	−2.459	1.9 × 10^−177^	0.361	0.302
	*EFEMP1*	EGF Containing Fibulin Extracellular Matrix Protein 1	−2.913	1.2 × 10^−81^	−2.784	1.1 × 10^−164^	0.322	0.673
	*FGFR2*	fibroblast growth factor receptor 2	−1.569	4.7 × 10^−107^	−3.144	1.7 × 10^−206^	0.329	0.675
	*SEMA3D*	Semaphorin 3D	−1.726	2.3 × 10^−77^	−5.453	9.2 × 10^−128^	1.88	3.10 × 10^−51^
Upregulated (14)	*CA2*	Carbonic Anhydrase II	1.749	3.5 × 10^−26^	4.437	3.6 × 10^−277^	0.271	0.024
	*CYP1A1*	Cytochrome P450, Family 1, Subfamily A, Polypeptide 1	1.604	4.0 × 10^−35^	4.102	1.2 × 10^−20^	−1.015	1.30 × 10^−7^
	*WNT11*	Wingless-Type MMTV Integration Site Family, Member 11	1.508	1.8 × 10^−10^	3.571	7.6 × 10^−29^	−1.042	0.16
	*LXN*	Latexin	2.559	3.9 × 10^−21^	3.087	0	0.207	0.735
	*TUBA4A*	Alpha-tubulin 4A isoform	1.924	3.1 × 10^−27^	2.347	8.1 × 10^−49^	−0.623	5.60 × 10^−15^
	*LINGO1*	Leucine Rich Repeat And Ig Domain Containing 1	1.541	2.7 × 10^−17^	2.065	7.9 × 10^−41^	−0.125	0.647
	*SLC2A6*	Solute Carrier Family 2 (Facilitated Glucose Transporter), Member 6	1.646	2.7 × 10^−22^	1.726	1.6 × 10^−50^	−1.182	5.40 × 10^−14^
	*TCIM*	Transcriptional And Immune Response Regulator	4.792	3.4 × 10^−191^	1.66	3.8 × 10^−20^	−0.234	0.003
	*DPYSL4*	Dihydropyrimidinase Like 4	3.423	4.70 × 10^−75^	1.643	3.6 × 10^−26^	−0.563	6.20 × 10^−4^
	*SCARA3*	Scavenger Receptor Class A Member 3	1.55	2.6 × 10^−23^	1.626	7.8 × 10^−23^	−0.897	3.20 × 10^−11^
	*SHC2*	SHC Adaptor Protein 2	3.194	9.5 × 10^−77^	1.608	1.2 × 10^−22^	−0.391	0.092
	*PLAC1*	Placenta-specific protein 1	1.533	2.2 × 10^−24^	1.592	2.2 × 10^−26^	0.035	0.631
	*PDIA2*	Protein Disulfide Isomerase Family A Member 2	1.848	4.1 × 10^−36^	1.578	1.1 × 10^−21^	−0.068	0.724
	*GALNT14*	Polypeptide N-Acetyl galactose aminyl transferase 14	2.015	7.7 × 10^−32^	1.519	1.2 × 10^−16^	−0.038	0.941

^a^ Expression levels of shared DEGs with |log_2_FC| ≥ 1.5, and *p*-adj < 0.01. ^b^ None of these DEGs showed a similar expression pattern in HeLa cell line analysis.

## Data Availability

The original contributions presented in the study are included in the article; further inquiries can be directed to the corresponding author. RNA-seq data can be found at https://www.ncbi.nlm.nih.gov/geo/query/acc.cgi?acc=GSE343535 (accessed on 12 August 2026), under accession number GSE343535.

## Supplementary Materials

The following supporting information can be downloaded at https://www.mdpi.com/article/10.3390/v18090934/s1, Figure S1: PCA plots and heatmaps of RNA-seq data analysis; Table S1: The concentration and purity of RNA samples subjected to RNA-seq analysis; Table S2: The primers used for the quantitative real-time PCR for validation of RNA-seq data of BLV-infected human cell lines; Table S3: Quantification of the total RNA reads, matched reads, and the properly matched read pairs in RNA samples analysis; Table S4: Detailed information on the top significant 20 DEGs of the three BLV-infected human cell lines analysis; Table S5: The top significant ten biological processes in GO analysis of the three BLV-infected human cell lines; Table S6: The top significant ten pathways in GO analysis of the three BLV-infected human cell lines.
