# Supplementary material for: Whole-Transcriptome Analysis of Three Human Cell Lines Stably Infected with Bovine Leukemia Virus (BLV) Reveals Novel Apoptosis-Associated Factors
Source: Viruses. 2026 Aug 27;18(9):934. doi: 10.3390/v18090934 (PMC13612058; doi:10.3390/v18090934)
Supplement: Supplementary file 1 [file viruses-18-00934-s001.zip › viruses-4465204-supplementary.pdf]

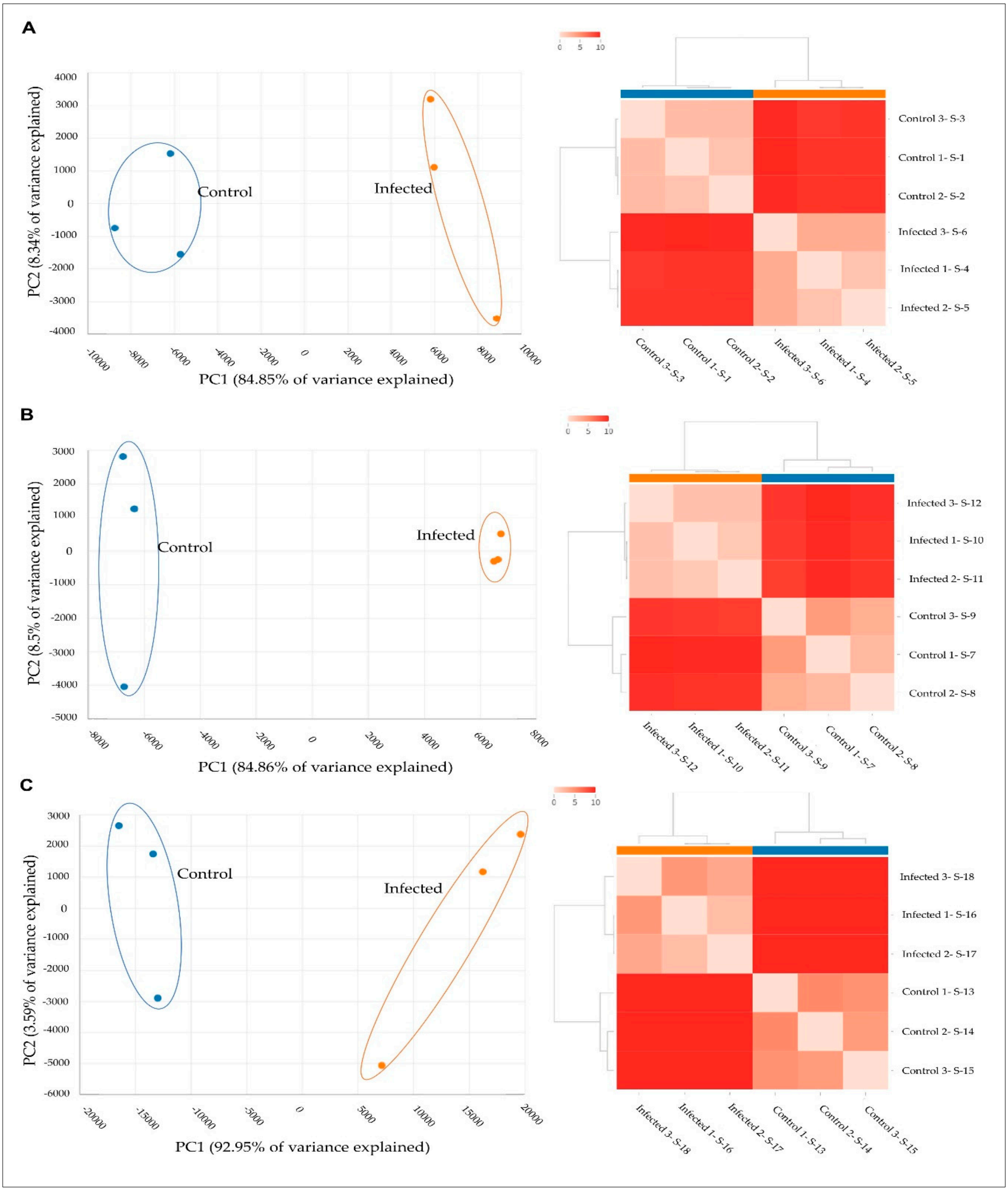

Figure S1. PCA plots and heatmaps of RNA-seq data analysis. PCA plots (left panels) of controls

(blue dots) vs. BLV-infected (brown dots) 293T (A), MCF7 (B), and HeLa (C) cell lines showing scatter plots of the samples along the first two principal components, and the heatmaps of expression similarity (right panels) showing the distances between each pair of samples.

Table S1. The concentration and purity of RNA samples subjected to RNA-seq analysis.

Sample	ID	ng/µl	A260/A280	A260/A280	Qubit ng/µl
239T	Control 1- S-1	919.2	2.12	2.22	1020
	Control 2- S-2	1436.2	2.13	2.27	1460
	Control 3- S-3	983.5	2.13	2.25	1020
239T-BLV	Infected 1- S-4	1105.2	2.11	2.26	1120
	Infected 2- S-5	1395.1	2.12	2.27	1400
	Infected 3- S-6	1206.2	2.12	2.20	1220
MCF7	Control 1- S-7	1561.5	2.14	2.21	1540
	Control 2- S-8	1664.7	2.13	2.20	1650
	Control 3- S-9	1103.6	2.12	2.21	1090
MCF7-BLV	Infected 1- S-10	1009.5	2.14	2.01	1000
	Infected 2- S-11	1230.7	2.15	2.27	1220
	Infected 3- S-12	1811.5	2.15	2.08	1800
HeLa	Control 1- S-13	1381	2.15	2.26	1380
	Control 2- S-14	1625	2.15	2.26	1620
	Control 3- S-15	1614	2.15	2.22	1610
HeLa-BLV	Infected 1- S-16	1676	2.15	2.26	1670
	Infected 2- S-17	2104	2.16	2.25	2100
	Infected 3- S-18	1577	2.16	2.25	1570

Table S2. The primers used for the quantitative real-time PCR for validation of RNA-seq data of BLV-infected human cell lines.

Gene	Primer		PCR Product length (bp)	Reference
	**Name**	**Sequences 5` to 3`**		
GAPDH	GAPDH-F	TTGGCTACAGCAACAGGGTG	161	[61]
	GAPDH-R	GGGGAGATTCAGTGTGGTGG		
RHOXF1P3	RHOXF1P3-F	GGATGTGAGTGAAGCCGAAG	190	This study
	RHOXF1P3-R	GTATCCCATTATTCCCCCGC		
NPNT	NPNT-F	GGACAATATCTGACAGTGTCGG	104	This study
	NPNT-R	GACAGGCACAGGTCCCCTGA		
EFEMP1	EFEMP1-F	CACGTACACGCAATGCACTG	157	[62]
	EFEMP1-R	CTGGGCTGTTTTCGGAAGGC		
FGFR2	FGFR2-F	GCCTCACCGGCCCATCCTCC	128	This study
	FGFR2-R	CCACGTGCTTGATCCACTGG		
SEMA3D	SEMA3D-F	CAACGCAGCCTGATAAACAAG	135	[63]
	SEMA3D-R	TCTTTCATCTCTTGTGGGGAGT		
CA2	CA2-F	CCTGAGCACTGGCATAAGGAC	142	[64]
	CA2-R	GGATCCTCAGGGAAGTTGCT		
CYP1A1	CYP1A1-F	TGAGTTCCTACCTGAACGGTTT	204	[65]
	CYP1A1-R	ATGGGGGTCATGTCCACCTTCA		
WNT11	WNT11-F	CTCGGAACTCGTCTATCTGCAG	105	[66]
	WNT11-R	CCGTTGGATGTCTTGTTGCAC		
LXN	LXN-F	TGCAGTTTCTTGTCCCGTTG	215	[67]
	LXN-R	GCCAGGCCCAATTAGATACCTC		

TUBA4A	TUBA4A-F	GGCAAGGAGATCATTGACCCAG	132	This study
	TUBA4A-R	CATCAGGAGTGAGGTGAAGCCA		
LINGO1	LINGO1-F	CTCCATGTTGCATGAGCTGCTC	147	This study
	LINGO1-R	GATTCCTCCAGTGTGGTCAGCT		
TCIM	TCIM-F	GAGAGGGAGGACAGGGAGAG	178	This study
	TCIM-R	CATCCATGTGGTGCACTTTTTC		
DPYSL4	DPYSL4-F	AGAAAACCTCATCGTCCCTGG	176	[68]
	DPYSL4-R	AGATCATGGTGGTTCCTCCT		
SHC2	SHC2-F	GCCCGGTCGGGCTCCGTCGG	180	This study
	SHC2-R	CCGCCGGGCCGCGGCCCAGG		
GALNT14	GALNT14-F	CACTGCTGGTGTATTGCACG	127	[69]
	GALNT14-R	CGGATCAGATGCGTAGGGGT		

Table S3. Quantification of the total RNA reads, matched reads, and the properly matched read pairs in RNA samples analysis.

Sample	ID	No. of total reads	No. of matched read pairs (%)	No. of properly matched read pairs (%)
239T	Control 1- S-1	37005589	30281735 (81.8%)	29019860 (95.8%)
	Control 2- S-2	35479418	29046516 (81.9%)	27716652 (95.4%)
	Control 3- S-3	30379588	24951996 (82.1%)	23903542 (95.8%)
239T-BLV	Infected 1- S-4	34998443	27042520 (77.3%)	25287463 (93.5%)
	Infected 2- S-5	33575103	26192896 (78%)	24837525 (94.8%)
	Infected 3- S-6	32699091	24767692 (75.7%)	23332067 (94.2%)
MCF7	Control 1- S-7	38970520	30826408 (79.1%)	29102950 (94.4%)
	Control 2- S-8	33968967	27247292 (80.2%)	25814149 (94.7%)
	Control 3- S-9	35558451	28646772 (80.6%)	27194291 (94.9%)
MCF7-BLV	Infected 1- S-10	35054867	28715491 (81.9%)	27262556 (94.9%)
	Infected 2- S-11	34239876	27931115 (81.6%)	26562322 (95.1%)
	Infected 3- S-12	36849933	30051827 (81.6%)	28750792 (95.7%)
HeLa	Control 1- S-13	32774381	27845681 (85%)	26995531 (96.9%)
	Control 2- S-14	41856017	35976614 (86%)	34919833 (97.1%)
	Control 3- S-15	53352055	46139500 (86.5%)	44944180 (97.4%)
HeLa-BLV	Infected 1- S-16	31503248	26564894 (84.3%)	25779876 (97%)
	Infected 2- S-17	39687003	33227057 (83.7%)	32248707 (97.1%)
	Infected 3- S-18	42270811	35644490 (84.3%)	34648650 (97.2%)

Table S4. Detailed information on the top significant 20 DEGs in the three BLV-infected human cell lines analysis.

Cell line	Gene symbol	Expression of control [a]	Expression of infected [a]	$\log_2$ FC	*p*-value	*p*-adj.
239T-BLV	*IGFBP5*	26.664	2.22	-3.103	0	0
	CCND2	6.224	48.93	2.789	0	0
	MAGEA10	14.181	232.352	3.942	0	0
	SERPINF1	148.989	10.159	-3.749	0	0
	IRS4	140.011	21.827	-2.627	0	0
	SETD1B	6.649	163.629	4.428	0	0
	HSPB8	2.51	72.885	4.396	0	0
	TSC22D3	742.677	115.12	-2.679	0	0
	DDR2	33.037	2.287	-3.372	0	0
	FAM84B	54.919	5.767	-3.047	0	0
	FAM133A	39.104	2.588	-3.482	0	0
	SHISA2	452.359	114.516	-1.973	0	0
	SMYD3	208.175	15.863	-3.633	0	0
	RYR2	0.811	21.529	3.637	0	0
	ID2	456.6	101.933	-2.152	2.1e-298	3.6e-295
	MAGEA9B	1.439	104.533	5.435	7.0e-291	1.1e-287
	ATF3	156.043	722.201	2.203	2.5e-288	3.8e-285
	BST2	99.722	7.5	-3.567	1.4e-247	2.0e-244
	NONO	675.769	256.024	-1.397	1.9e-238	2.5e-235
	SLC16A2	14.08	0.691	-3.156	4.2e-217	5.4e-214
MCF7-BLV	*SLC7A2*	330.266	53.704	-2.598	0	0
	CALCR	192.714	36.755	-2.359	0	0

	LXN	40.582	352.403	3.087	0	0
	LAMB1	50.775	2.799	-3.768	0	0
	CPD	6.942	41.126	2.407	0	0
	PMP22	171.414	27.181	-2.613	0	0
	COL12A1	67.064	2.048	-4.481	0	0
	SSFA2	347.151	60.147	-2.509	0	0
	HMCN1	57.355	3.658	-3.647	0	0
	AFAP1L2	62.744	5.201	-3.362	0	0
	SOX2	6.991	64.874	3.043	0	0
	HLA-DRB1	2.371	215.768	6.007	0	0
	AJUBA	103.923	19.37	-2.365	1.4e-288	2.7e-285
	SIGLEC15	1.895	37.103	3.719	1.6e-285	2.8e-282
	CA2	1.945	62.782	4.437	2.2e-280	3.6e-277
	SLC4A10	30.006	3.452	-2.8	2.8e-267	4.4e-264
	ST3GAL1	10.173	45.717	2.064	3.0e-262	4.5e-259
	INHBA	0.541	16.039	3.467	3.0e-260	4.2e-257
	SCNN1A	5.656	45.458	2.803	1.2e-259	1.6e-256
	EVL	513.658	133.16	-1.94	3.9e-249	5.0e-246
HeLa-BLV	*YWHAE*	277.99	54.074	-2.341	4.5e-122	1.1e-117
	PTPRR	21.162	2.361	-2.721	1.9e-96	2.3e-92
	LUM	40.749	272.102	2.71	4.5e-81	3.6e-77
	PDK4	10.654	93.213	3.015	8.6e-74	5.1e-70
	SLC43A3	82.946	24.665	-1.71	5.7e-73	2.7e-69
	ALDOA	58.426	7.147	-2.867	3.0e-68	1.2e-64
	GSTA4	10.086	57.991	2.412	1.1e-67	3.7e-64
	MRPL28	131.779	47.931	-1.44	4.8e-64	1.4e-60
	PTGES	56.73	16.634	-1.711	3.2e-62	8.5e-59

AC034102.2	0.372	248.58	7.507	1.0e-61	2.4e-58
ABLIM2	19.025	3.194	-2.255	8.0e-61	1.7e-57
PIK3CD	15.261	2.705	-2.134	5.4e-59	1.1e-55
C5AR2	9.225	1.406	-2.087	1.1e-57	2.0e-54
PLEKHA6	6.975	0.682	-2.246	1.7e-57	2.9e-54
PRKAR1B	47.721	12.799	-1.82	1.1e-56	1.8e-53
NPTX1	54.272	14.696	-1.816	1.6e-56	2.4e-53
SEMA3D	1.172	6.994	1.88	2.2e-54	3.1e-51
HDAC9	2.186	11.969	2.025	4.8e-54	6.3e-51
UBE2S	275.602	77.583	-1.816	1.7e-49	2.1e-46
ABCC2	7.182	2.545	-1.207	2.4e-48	2.9e-45

[a] The mean expression of each gene as calculated with the TPMs values.

Table S5. The top significant ten biological processes in GO analysis of the three BLV-infected human cell lines.

Cell line	GO-Term	Category	No. of DEGs in category	Percentage of DEGs annotated	*p*-value
293T-BLV	① transcription, DNA-templated	Process	137	10.804	7.7e-11
	② regulation of transcription by RNA polymerase II	Process	74	7.741	8.4e-11
	③ apoptotic process	Process	9	0.959	7.0e-7
	④ DNA repair	Process	15	1.605	1.9e-6
	⑤ cellular response to DNA damage stimulus	Process	15	1.605	2.1e-5
	⑥ regulation of transcription, DNA-templated	Process	53	5.584	2.5e-5
	⑦ protein transport	Process	24	2.584	2.5e-5
	⑧ SRP-dependent cotranslational protein targeting to membrane	Process	7	0.771	4.8e-5
	⑨ protein phosphorylation	Process	29	3.053	4.9e-5
	⑩ translation	Process	13	1.417	6.9e-5
MCF7-BLV	① protein transport	Process	22	2.844	3.7e-8
	② ephrin receptor signaling pathway	Process	7	0.761	4.1e-8
	③ cell migration	Process	13	1.634	1.2e-7
	④ cell cycle	Process	17	2.146	3.9e-7
	⑤ actin cytoskeleton organization	Process	9	1.16	1.3e-6

	⑥ positive regulation of cell migration	Process	12	1.497	1.3e-6
	⑦ positive regulation of apoptotic process	Process	18	2.27	1.4e-6
	⑧ nuclear-transcribed mRNA catabolic process, nonsense-mediated decay	Process	8	1.098	1.4e-6
	⑨ apoptotic process	Process	29	3.73	1.7e-6
	⑩ semaphorin-plexin signaling pathway	Process	3	0.374	5.0e-6
HeLa-BLV	① cell division	Process	26	2.838	2.3e-14
	② cell cycle	Process	21	2.226	2.7e-11
	③ viral process	Process	25	2.637	3.2e-11
	④ transcription, DNA-templated	Process	101	10.75	2.4e-10
	⑤ protein ubiquitination	Process	31	3.291	6.9e-10
	⑥ DNA repair	Process	16	1.783	7.1e-10
	⑦ protein transport	Process	26	2.806	2.4e-9
	⑧ ciliary basal body-plasma membrane docking	Process	8	0.876	3.9e-9
	⑨ cellular response to DNA damage stimulus	Process	16	1.783	1.4e-8
	⑩ protein phosphorylation	Process	31	3.323	2.4e-8

Table S6. The top significant ten pathways in GO analysis of the three BLV-infected human cell lines.

Cell line	Pathway	Database	No. of DEGs in category	Percentage of DEGs annotated	*p*-value
293T-BLV	① Gene Expression	REACTOME	116	18.576	3.2e-16
	② Generic Transcription Pathway	REACTOME	56	8.985	1.1e-10
	③ Organelle biogenesis and maintenance	REACTOME	24	3.855	6.2e-9
	④ Mitochondrial tRNA aminoacylation	REACTOME	4	0.717	1.1e-5
	⑤ Ribosome	KEGG	10	1.705	1.3e-5
	⑥ Membrane Trafficking	REACTOME	39	6.229	3.3e-5
	⑦ Apoptosis	KEGG	7	1.139	4.5e-5
	⑧ Transcriptional Regulation by TP53	REACTOME	24	3.887	5.2e-5
	⑨ Axon guidance	REACTOME	34	5.528	7.3e-5
	⑩ Regulation of cholesterol biosynthesis by SREBP (SREBF)	REACTOME	5	0.896	7.5e-5
MCF7-BLV	① Axon guidance	REACTOME	31	5.87	6.4e-8
	② EPH-Ephrin signaling	REACTOME	7	1.34	9.5e-8
	③ Endocytosis	KEGG	16	3.114	9.7e-8
	④ Membrane Trafficking	REACTOME	34	6.493	2.7e-6
	⑤ Gene Expression	REACTOME	95	18.026	3.0e-6
	⑥ Metabolism of proteins	REACTOME	84	15.912	3.1e-6
	⑦ Exon junction complex (EJC)	KEGG	9	1.831	5.0e-6
	⑧ Apoptosis	KEGG	8	1.513	7.0e-6
	⑨ MicroRNAs in cancer	KEGG	9	1.831	1.4e-5

	⑩ Disease	REACTOME	46	8.739	1.9e-5
HeLa-BLV	① Cell Cycle, Mitotic	REACTOME	38	6.192	1.0e-17
	② Cell Cycle	REACTOME	46	7.354	1.2e-17
	③ Gene Expression	REACTOME	120	19.15	4.4e-17
	④ Membrane Trafficking	REACTOME	44	7.068	2.4e-16
	⑤ Vesicle-mediated transport	REACTOME	46	7.323	2.2e-13
	⑥ Organelle biogenesis and maintenance	REACTOME	25	4.123	1.3e-12
	⑦ RHO GTPases Activate Formins	REACTOME	10	1.624	1.9e-12
	⑧ Mitotic Prometaphase	REACTOME	9	1.528	1.0e-11
	⑨ Mitotic Metaphase and Anaphase	REACTOME	14	2.372	1.2e-11
	⑩ Mitotic Anaphase	REACTOME	14	2.356	1.8e-11
